# Low-temperature cold plasma promotes wound healing by inhibiting skin inflammation and improving skin microbiome

**DOI:** 10.3389/fbioe.2025.1511259

**Published:** 2025-02-20

**Authors:** Jie Zhou, Zengkun Sun, Xiaoru Wang, Shouguo Wang, Wen Jiang, Dongqi Tang, Tao Xia, Fang Xiao

**Affiliations:** ^1^ School of Bioengineering, Qilu University of Technology (Shandong Academy of Science), Jinan, Shandong, China; ^2^ State Key Laboratory of Biobased Material and Green Papermaking, Qilu University of Technology (Shandong Academy of Science), Jinan, Shandong, China; ^3^ Academy of Advanced Interdisciplinary Studies, Qilu University of Technology (Shandong Academy of Science), Jinan, Shandong, China; ^4^ Beijing Zhongsu Titanium Alloy Vacuum Plasma Technology Research Institute, Beijing, China; ^5^ Center for Gene and Immunotherapy, Multidisciplinary Innovation Center for Nephrology, The Second Hospital, Cheeloo College of Medicine, Shandong University, Jinan, Shandong, China; ^6^ Department of Gerontology, The Second Hospital, Cheeloo College of Medicine, Shandong University, Jinan, Shandong, China

**Keywords:** low-temperature cold plasma, wound healing, inflammation, senescence-associated secretory phenotype, tissue repair factors, cell proliferation and apoptosis, skin microbiome

## Abstract

Wound healing includes four consecutive and overlapping stages of hemostasis, inflammation, proliferation, and remodeling. Factors such as aging, infection, and chronic diseases can lead to chronic wounds and delayed healing. Low-temperature cold plasma (LTCP) is an emerging physical therapy for wound healing, characterized by its safety, environmental friendliness, and ease of operation. This study utilized a self-developed LTCP device to investigate its biological effects and mechanisms on wound healing in adult and elderly mice. Histopathological studies found that LTCP significantly accelerated the healing rate of skin wounds in mice, with particularly pronounced effects in elderly mice. LTCP can markedly inhibit the expression of pro-inflammatory cytokines (*TNF-α*, *IL-6*, *IL-1β*) and senescence-associated secretory phenotype factors (*MMP-3*, *MMP-9*), while significantly increasing the expression of tissue repair-related factors, such as *VEGF*, *bFGF*, *TGF-β*, *COL-I*, and *α-SMA*. It also regulated the expression of genes related to cell proliferation and migration (*Aqp5*, *Spint1*), inflammation response (*Nlrp3*, *Icam1*), and angiogenesis (*Ptx3*, *Thbs1*), promoting cell proliferation and inhibit apoptosis. Furthermore, LTCP treatment reduced the relative abundance of harmful bacteria such as *Delftia*, *Stenotrophomonas*, *Enterococcus*, and *Enterobacter* in skin wounds, while increasing the relative abundance of beneficial bacteria such as *Muribaculaceae*, *Acinetobacter*, *Lachnospiraceae NK4A136_group*, and *un_f__Lachnospiraceae*, thereby improving the microbial community structure of skin wounds. These research findings are of significant implications for understanding the mechanism of skin wound healing, as well as for the treatment and clinical applications of skin wounds, especially aging skin.

## 1 Introduction

The skin is the largest organ of the human body, primarily composed of the epidermis, dermis, subcutaneous tissue, and other skin appendages, such as hair follicles, sweat glands, and sebaceous glands (Hosseini et al., 2022). Accounting for 15% of body weight, the skin plays a vital role in thermoregulation, sensation, and fluid balance (Liang et al., 2022). It also serves as a physical barrier that protects internal organs from external environmental factors (Moeini et al., 2020). Skin damage refers to the injury of the skin tissue structure or integrity caused by various intrinsic pathological factors and extrinsic mechanical factors (Zhou et al., 2023). Wounds are classified as either acute or chronic based on the healing duration (Dubey et al., 2022). Acute wounds, which result from factors such as radiation, extreme temperature changes, or exposure to chemicals (Moeini et al., 2020), typically heal on their own within 2–12 weeks (Ji et al., 2022). In contrast, chronic wounds usually require a longer healing time (Moeini et al., 2020). Numerous factors contribute to chronic wounds, including aging, infection, chronic diseases, vascular insufficiency, diabetes, malnutrition, and edema. Common types of chronic wounds include venous ulcers, arterial ulcers, pressure sores, and diabetic foot ulcers (Zhao et al., 2016; Rezaie et al., 2019). With aging, the skin becomes fragile, atrophic, dry, and loses elasticity, and its immune components also undergo alterations, resulting in diminished adaptive capacity of the skin’s immune function. Aged skin is more prone to injury and more slowly to heal after damage (Ding et al., 2021). It is reported that approximately 1%–2% of the population in developed countries suffers from chronic wounds, and 4%–10% of diabetic patients experience chronic bacterial infections wounds each year globally. Due to the long and painful healing period of chronic wounds, it not only effects patients’ quality of life but also contributes to high morbidity and mortality rates. The care and treatment of chronic wounds account for approximately 2%–4% of the global healthcare budget, and are projected to reach $18.7 billion by 2027 (Wang Y. et al., 2021; Yu et al., 2022; Manchanda et al., 2023).

Skin wound healing is a highly complex dynamic process that encompasses four continuous and overlapping phases: hemostasis, inflammation, proliferation, and remodeling (Knoedler et al., 2023). This process involves interactions between multiple cell populations, soluble mediators and cytokines (Liang et al., 2022). The hemostasis phase begins immediately after injury, forming fibrin clot and vasoconstriction. Subsequently, inflammatory cells such as neutrophils, macrophages, and lymphocytes begin to migrate towards the wound, initiating the inflammatory phase of wound healing (Gharbia et al., 2023). During the proliferation stage, new granulation tissue grows in the wound area through epithelialization, forming a new extracellular matrix (ECM). The final phase of wound healing is remodeling, during which the composition of the matrix changes, with type III collagen gradually being replaced by type I collagen, resulting in increased tensile strength of the newly formed tissue (Moeini et al., 2020).

Currently, there are many methods available for wound treatment in clinical practice, such as medications, antibiotics, wound dressings, growth factors, stem cell transplants, stem cell sprays, negative pressure therapy, hyperbaric oxygen, electrical stimulation, and skin grafting (Bi et al., 2019; Zhang et al., 2021; Kou et al., 2023). These treatments, which are based on different mechanisms of skin wound healing, are widely applied in clinical practice. However, they suffer from limitations such as antibiotic resistance, operational complexity, high cost, high time cost, immune rejection, microbial infections, and low cure rates (Rezaie et al., 2019; Luo et al., 2021; Dubey et al., 2022; Yu et al., 2022). Wound treatment, especially for chronic wounds, still faces significant challenges. In recent years, Low-Temperature Cold Plasma (LTCP) has emerged as a novel wound healing therapy. It not only offers advantages such as safety, environmental friendliness, and ease of operate but also shows good efficacy in promoting wound healing (Boekema et al., 2021; Sedik et al., 2023). However, the biological effects of LTCP on wound healing are highly influenced by its operational parameters.

Plasma is an ionized gas and the fourth state of matter. It contains a high concentration of charged particles (OH^−^, H_2_O^+^, and electrons), active chemical substances (reactive oxygen species (ROS) and reactive nitrogen species (RNS)), excited molecules, and ultraviolet photons (UVB, UVC) (Niedźwiedź et al., 2019). In atmospheric pressure plasma, the interaction between plasma and air leads to the partial dissociation and ionization of surrounding O_2_, N_2_, and H_2_O, resulting in the generation of reactive chemical species capable of inducing specific intracellular reactions. These species include reactive oxygen species (ROS), such as ozone (O_3_), hydrogen peroxide (H_2_O_2_), hydroxyl (OH), hydroxyl radicals (·OH), superoxide (O_2_^−^·), and singlet oxygen (^1^O_2_), as well as reactive nitrogen species (RNS), such as nitric oxide (NO), nitrogen dioxide (NO_2_), dinitrogen trioxide (N_2_O_3_), dinitrogen tetroxide (N_2_O_4_), nitrous oxide (N_2_O), and peroxynitrite (ONOO⁻), among others (Ishaq et al., 2015; Karthik et al., 2023). According to its temperature, it can be classified into standard (“thermal”) plasma at 4,000–5000 K and low-temperature (“cold” or “non-thermal”) plasma at 30°C–50°C (Martusevich et al., 2022). In the biomedical field, LTCP has a wide range of applications in bacterial inactivation, oral medicine, tumor therapy, skin disease treatment, and wound healing (Nguyen et al., 2019; Duarte and Panariello, 2020; Choi et al., 2021). Clinically, LTCP has been effective in promoting the healing of various types of superficial skin wounds, such as diabetic foot ulcers, chronic eczema, giant genital warts, and gangrenous pyoderma, with complete wound healing observed in patients without adverse reactions following plasma treatment (Gao et al., 2019). The LTCP devices used in clinical and experimental settings can be categorized into three types: plasma based on direct discharge such as dielectric barrier discharge, plasma based on indirect discharge such as plasma jets and plasma pens, and hybrid plasma devices (Hoffmann et al., 2013; Isbary et al., 2013). The discharge principle, power, effective area and other parameters of various types of plasma devices vary, and they also present limitations such as poor portability, difficulty in operation, and restricted usage in certain environments. Additionally, there is a lack of systematic research on the biological mechanisms of LTCP in promoting wound healing, especially its impact on the microbial community of skin wounds.

This study used a self-developed small LTCP device to verify its effect on skin wound healing in adult and elderly mice and explore its mechanism of action, providing new ideas, methods, and data support for the treatment of skin wounds and further clinical research.

## 2 Materials and methods

### 2.1 Development and working principle of LTCP devices

**FIGURE 1:**
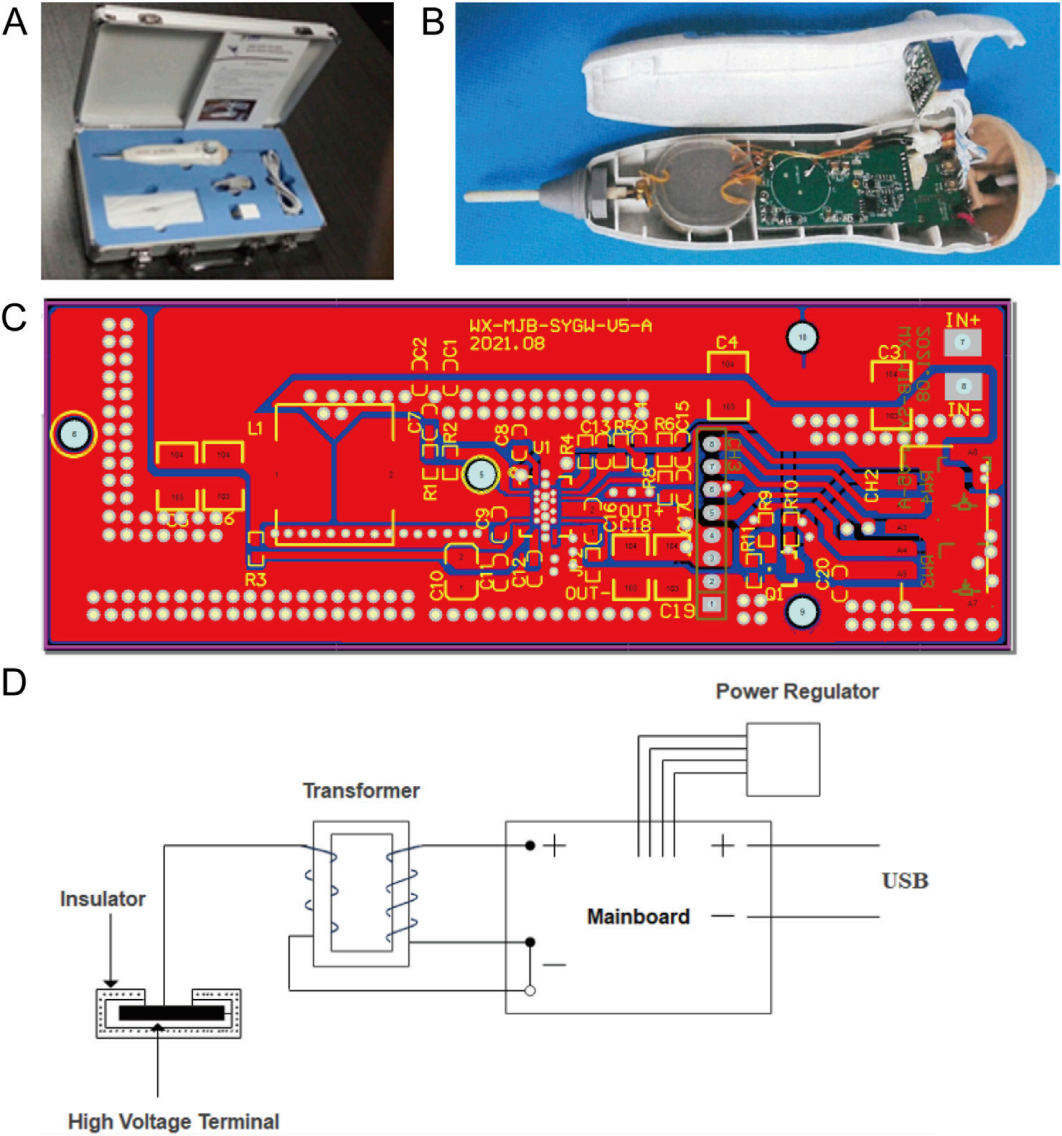
Schematic diagram of the LTCP device and its working principle. **(A)** A single electrode dielectric barrier LTCP sterilization pen that integrates battery drive and USB interface. **(B)** Internal structure diagram of the LTCP device. **(C)** Battery power circuit diagram. **(D)** Schematic representation of the working principle of the LTCP device.

The LTCP device developed in this study is a single electrode dielectric barrier LTCP sterilization pen that integrated battery drive and USB interface (5 V working voltage) (as shown in Figure 1). Unlike plasma discharge generated by direct connection to a 220 V power source, the dielectric barrier LTCP discharge instruments emit stronger electromagnetic radiation to the outside. On the basis of dielectric barrier discharge, the human body is used as another electrode to achieve the sterilization application of single electrode dielectric barrier discharge on the human body. At the same time, the discharge electrode can be plugged and replaced, avoiding cross infection during use.

The mainboard of the LTCP equipment is powered by a power supply, which converted the input current into a pulse current and outputted it to the transformer. The output power of the mainboard can be adjusted through a power regulator. Then the output current is passed through a transformer to increase the voltage, with the high-voltage end of the secondary coil suspended. The discharge electrode is covered with insulating material, and dielectric discharge occurs when the low-voltage point approaches. When discharging, it ionizes the surrounding air and generated plasma. The effective diameter of the plasma nozzle is 100 mm, the nozzle temperature is between 40°C and 70°C, and the effective plasma action area for living organisms is greater than 30 cm^2^. The key components of the plasma generator can operate continuously and stably for over 200 h, with a rated power of 0–10 kW.

We referred to previous methods to conduct a preliminary detection of the total reactive oxygen and nitrogen species (RONS) produced by the plasma device (Gan et al., 2019). Add 1 mL of fresh DMEM medium to a 48-well plate, place the LTCP into the wells containing the medium, and treat for 2 min to prepare plasma-activated medium (PAM). Use untreated medium as a blank control. Dilute the DCFH-DA (Sigma-Aldrich, United States) stock solution with the medium to a final concentration of 100 µM. Then, treat the medium containing the DCFH-DA probe with LTCP following the same method used to prepare PAM. After treatment, measure the fluorescence intensity using a multifunctional microplate reader (Flexstation 3, Molecular Devices, United States). The results indicate that the concentration of representative active substances generated by the plasma is no less than 200 ppm.

### 2.2 Experimental mice and wound healing experiments

Two-month-old male C57BL/6J mice were purchased from Beijing Vital River Laboratory Animal Technology. Co., Ltd (Beijing, China, Production License No: SCXK(Beijing) 2021–0006), while eighteen-month-old male C57BL/6J mice were purchased from Shanghai Model Organisms Center, Inc (Shanghai, China, Production License No: SYXK (Shanghai) 2023–0005). During the experiment, the mice were kept in a well-ventilated environment with a room temperature of 23°C ± 2°C, relative humidity of 45%–65%, and a 12-hour light-dark cycle, with free access to food and water. Each mouse was kept in a separate cage to prevent mutual biting and to ensure the integrity of the skin on their backs.

After 1 week of acclimatization, the mice were anesthetized by intraperitoneal injection of 10% (w/v) chloral hydrate. The hair on their dorsal skin was removed using an electric hair clipper, and full-thickness skin excision circular wound were created using a 6 mm sterile biopsy puncture device (Archer et al., 2020). Ten two-month-old mice were randomly divided into two groups (n = 5): the Young Control Group (YC) and the Young LTCP Treatment Group (YP). Similarly, ten eighteen-month-old mice were randomly assigned to two groups (n = 5): the Old Control Group (OC) and the Old LTCP Treatment Group (OP). The wounds of the YC and OC groups were left untreated, while the YP and OP groups were gently touched back and forth at the wound site using the LTCP device after surgery. They received LTCP treatment once daily for a week, followed by every other day treatments after 1 week, with each session lasting 2 min for a total of 12 days.

The day of the biopsy procedure was designated as Day 0. During the postoperative period from Days 0–12, the wound area of each group of mice was monitored and measured using digital calipers and photographs, allowing to plot a wound area change curve. On the third postoperative day, the mice in each group were anesthetized via intraperitoneal injection of 10% (w/v) chloral hydrate, and skin samples from the wound area on the back of the mice were collected. One part of the sample was immediately treated with liquid nitrogen and stored at −80°C, while another part was fixed in 4% (w/v) paraformaldehyde.

All animal experiments in this study were approved by the Ethics Committee of the Experimental Animal Center of the Second Hospital of Shandong University (Jinan, China) and strictly adhered to the “Regulations for the Administration of Laboratory Animals” issued by the Ministry of Science and Technology of the People’s Republic of China.

### 2.3 Histopathological observation

Skin tissues fixed in 4% (w/v) paraformaldehyde were embedded in paraffin and sectioned into 4 μm slices using a microtome (RM2016, Shanghai Leica Instruments Co., Ltd., Shanghai, China). The sections were stained with hematoxylin and eosin (H&E) and subsequently examined under an optical microscope (Nikon, Tokyo, Japan) for histopathological observation and imaging.

### 2.4 Immunofluorescence staining and observation

Skin tissues fixed in 4% (w/v) paraformaldehyde were embedded in paraffin and sectioned into 4 μm slices using a microtome (RM2016, Shanghai Leica Instruments Co., Ltd., Shanghai, China). After dewaxing and gradient ethanol solution (95%–75% (v/v)) hydration, the slices were blocked with 3% (w/v) BSA for 30 min. Primary antibody (Anti-Ki67 Mouse mAb, Wuhan Service Biotechnology Co., Ltd., Hubei, China. Dilution ratio 1:200.) was applied and incubated overnight at 4°C. Following this, the sections were washed three times with PBS buffer (pH 7.4) by shaking on a decolorization shaker. Subsequently, the sections were incubated with the secondary antibody (Alexa Fluor 488 Goat Anti-Mouse IgG, Wuhan Service Biotechnology Co., Ltd., Hubei, China. Dilution ratio 1:400) at 37°C for 50 min. After three additional washes with PBS buffer (pH 7.4) by shaking on the decolorization shaker, DAPI staining solution was added for nuclear counterstaining for 10 min at room temperature. The sections were then washed three times with PBS buffer (pH 7.4) and treated with the autofluorescence quencher B solution for 5 min, followed by rinsing with running water for 10 min, and then sealed with anti-fluorescence quencher. Finally, the sections were observed and photographed under an optical microscope (Nikon, Tokyo, Japan).

### 2.5 TUNEL staining and observation

Skin samples from the wound sites on the backs of different mice were collected on postoperative days 0, 3, 7, and 10. The tissues were fixed in 4% (w/v) paraformaldehyde, embedded in paraffin, and sectioned into 4 μm slices using a microtome (RM2016, Shanghai Leica Instruments Ltd., Shanghai, China). The sections were deparaffinized with xylene and hydrated using a gradient ethanol solution (95%–75% (v/v)). The TUNEL assay kit (Wuhan Service Biotechnology Co., Ltd., Hubei, China) was employed to analyze cell apoptosis. The TUNEL reaction mixture (Recombinant TdT Enzyme, TMR-5-dUTP Labeling Mix, Equilibration Buffer, Proteinase K) was fluorescence stained for 1 hour at 37°C, followed by the addition of DAPI staining solution for nuclear counterstaining for 10 min at room temperature. The sections were washed three times with PBS buffer (pH 7.4), lightly dried, and then sealed with anti-fluorescence quenching sealing agent. Finally, the sections were observed and photographed under an optical microscope (Nikon, Tokyo, Japan). Use ImageJ software to analyze images.

### 2.6 Skin tissue transcriptome analysis

Total RNA was extracted from skin tissue using Trizol reagent kit (Takara, Japan). Subsequently, a cDNA library was constructed and sequenced using the DNBSEQ high-throughput sequencing platform (DNBSEQ-T7, Shenzhen, China) and for subsequent analysis.

### 2.7 Quantitative real-time PCR detection of mRNA expression of skin tissue-related genes

Total RNA was extracted from skin tissue using Trizol reagent kit (Takara, Japan), and complementary DNA (cDNA) was synthesized using PrimeScript™ RT reagent Kit with gDNA Eraser (Takara, Japan). Quantitative real-time PCR (qRT-PCR) was performed for the genes *TNF-α*, *IL-6*, *IL-1β*, *MMP-3*, *MMP-9*, *VEGF*, *bFGF*, *TGF-β*, *COL-I*, *α-SMA*, *Aqp5*, *Spint1*, *Nlrp3*, *Icam1*, *Ptx3*, and *Thbs1*, using SYBR^®^ Premix Ex Taq™ II (Takara, Japan) and QuantStudio 3 Real-Time PCR Systems (Thermo Fisher, United States). The primer sequences were listed in Table 1. The qRT-PCR reaction conditions were as follows: pre denaturation at 95°C for 30 s, 40 cycles, 95°C for 5 s, and 60°C for 34 s. *GADPH* was used as the reference gene and data were analyzed using the 2^−ΔΔCT^ method.

**TABLE 1:** Sequences of primers for qRT-PCR.

Gene	Forward primer (5′to3′)	Reverse primer (5′to3′)
*GADPH*	TGT​GTC​CGT​CGT​GGA​TCT​GA	TTG​CTG​TTG​AAG​TCG​CAG​GAG
*TNF-α*	GAC​GTG​GAA​CTG​GCA​GAA​GAG	GCC​ACA​AGC​AGG​AAT​GAG​AAG
*IL-6*	ACA​AAG​CCA​GAG​TCC​TTC​AGA​G	GGCAGAGGGGTTGACTT
*IL-1β*	TCC​AGG​ATG​AGG​ACA​TGA​GCA	GAA​CGT​CAC​ACA​CCA​GCA​GGT
*MMP-3*	TGC​ATG​ACA​GTG​CAA​GGG​AT	ACA​CCA​CAC​CTG​GGC​TTA​TG
*MMP-9*	AAC​CTC​CAA​CCT​CAC​GGA​CA	TTT​GGA​ATC​GAC​CCA​CGT​CT
*VEGF*	GCA​GGG​GAC​AGA​GGG​ACT​TG	GAG​GCC​ATC​GCT​GCA​CTC​A
*bFGF*	GTC​AAA​CTA​CAG​CTC​CAA​GCA​GAA	AGG​TAC​CGG​TTC​GCA​CAC​A
*TGF-β*	CTC​CCG​TGG​CTT​CTA​GTG​C	GCC​TTA​GTT​TGG​ACA​GGA​TCT​G
*COL-I*	GAC​AGG​CGA​ACA​AGG​TGA​CAG​AG	CAG​GAG​AAC​CAG​GAG​AAC​CAG​GAG
*α-SMA*	GTC​CCA​GAC​ATC​AGG​GAG​TAA	TCG​GAT​ACT​TCA​GCG​TCA​GGA
*Aqp5*	TGG​AGC​AGG​CAT​CCT​GTA​CT	CGG​TGA​AGT​AGA​TCC​CCA​CAA
*Spint1*	GCT​CTG​TGT​TGG​GGT​CAC​A	CAA​AGG​AGC​CAT​ACG​CCG​A
*Nlrp3*	AGG​CTG​CTA​TCT​GGA​GGA​ACT	CTT​TCT​CGG​GCG​GGT​AAT​CT
*Icam1*	GCC​TCC​GGA​CTT​TCG​ATC​TT	TGT​TTG​TGC​TCT​CCT​GGG​TC
*Ptx3*	CAGGAGAGCCGTGACGC	ATT​GCT​GTT​TCA​CAA​CCT​GCG
*Thbs1*	TGC​AGG​ACA​GCA​TCC​GAA​AA	GGT​AAC​CGA​GTT​CTG​GCA​GT

### 2.8 16S rRNA sequencing to detect changes of skin microbial communities

**FIGURE 2:**
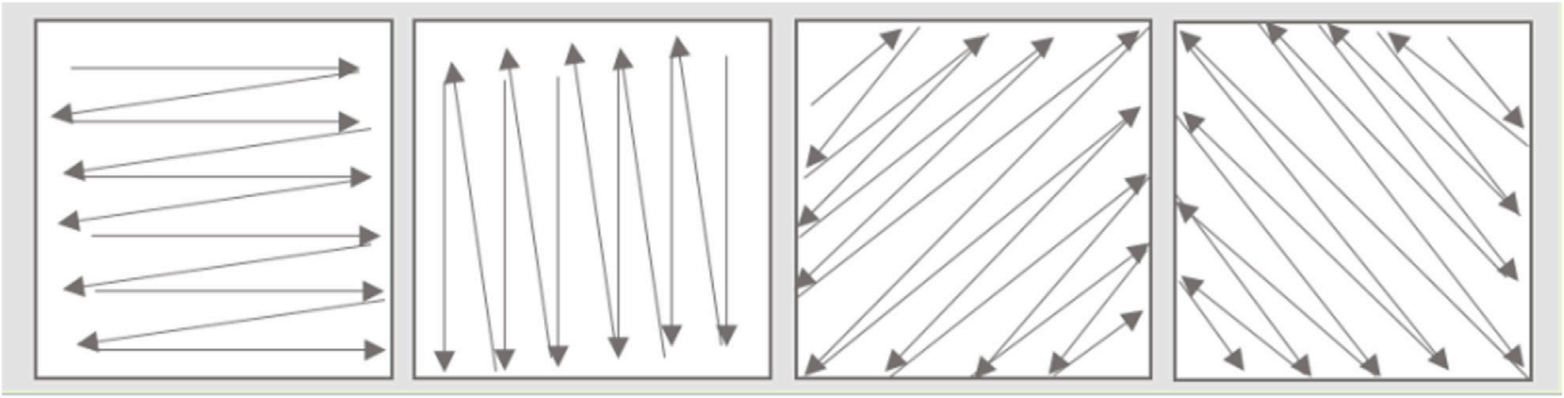
Directions of microbial sample collection.

After a 1-week acclimatization period, four 2-month-old and four 18-month-old male mice were anesthetized, and their back hair was removed using an electric shaver. Using a sterile swab moistened with sampling solution (0.15 M NaCl and 0.1% (w/v) Triton X-100) to gently swab the area on the back skin where the wound was to be created. Following sample collection, the swab heads were snapped off and placed in sterile 2 mL EP tubes. Subsequently, a full-thickness skin excision wound measuring 1 cm × 1 cm was created on the back of each mouse. Samples were collected from the wound site 24 h post-wounding. Then the wounds were treated with LTCP for 2 min, and samples were collected again 24 h after treatment. Immediately after each sample collection, the samples were treated with liquid nitrogen and stored at −80°C. Placing a soaked swab in the sampling environment as a blank control during each collection (leave it in the environment for 10 min). Wipe the skin at the sampling site in the direction shown in Figure 2, wiping more than 10 times in each direction, rotating the swab head 90° for each direction collected. The collected samples were subjected to 16S rRNA sequencing using the Illumina high-throughput sequencer (Illumina NovaSeq 6,000, United States) for subsequent analysis.

### 2.9 Statistical analysis

Statistical analysis and plotting were conducted using GraphPad Prism 9. Comparisons among multiple groups were performed using one-way analysis of variance (ANOVA), while the graphs of wound area changes and The Apoptotic Index of TUNEL Staining were analyzed using two-way ANOVA. All data are presented as mean ± standard deviation (SD), and a p-value of p < 0.05 was considered statistically significant.

## 3 Results

### 3.1 The effect of LTCP on skin wound healing

**FIGURE 3:**
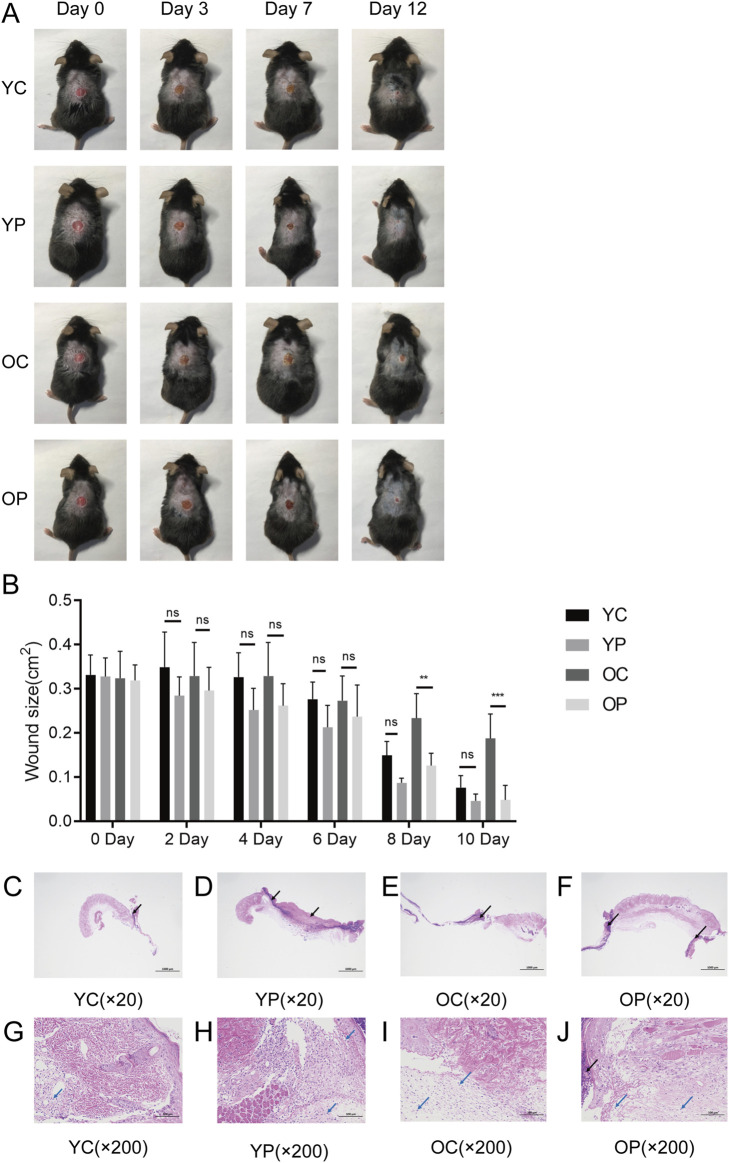
The Effect of LTCP on Skin Wound Healing. **(A)** Wound healing status of each group at post-operative days 0, 3, 7, and 12 (n = 5). **(B)** Wound area for each group from post-operative days 0–10 (n = 5). **(C–J)** Histological changes in wounds of each group on post-operative day 3 (n = 3). **(C–F)** HE staining, scale bar = 1,000 μm. **(G–J)** HE staining, scale bar = 100 μm. Data are expressed as mean ± SD. **P < 0.01, ***P < 0.001. P values were calculated using Two-Way ANOVA.

We selected 2-month-old and 18-month-old mice and established skin injury models on their backs. The wound area of the mice in each group were monitored and measured using digital calipers and photographs from day 0 to day 12 post-surgery. As illustrated in Figures 3A,B, the wound healing speed in the OC group mice was significantly delayed compared to the other three groups, which was consistent with the previous reported (Li et al., 2023). Compared with the YC and OC groups, the YP and OP groups exhibited a marked acceleration in wound healing speed after day 7 post-surgery. On day 3 post-surgery, wound skin tissue samples were collected from each group of mice. Through histopathological analysis, it was observed that the YP and OP groups of mice had better wound tissue pathology than the YC and OC groups (Figures 3C–J). Compared with the YC and OC groups, the skin tissue necrosis area in the YP and OP groups decreased (black arrow), with more granulation tissue and fibroblast proliferation visible, accompanied by less lymphocyte and granulocyte infiltration (blue arrow). The above results indicated that LTCP could promote skin wound healing and significantly reduce the healing time for wounds in aged skin.

### 3.2 The effect of LTCP on the expression of inflammatory factors and senescence-associated secretory phenotype factors

**FIGURE 4:**
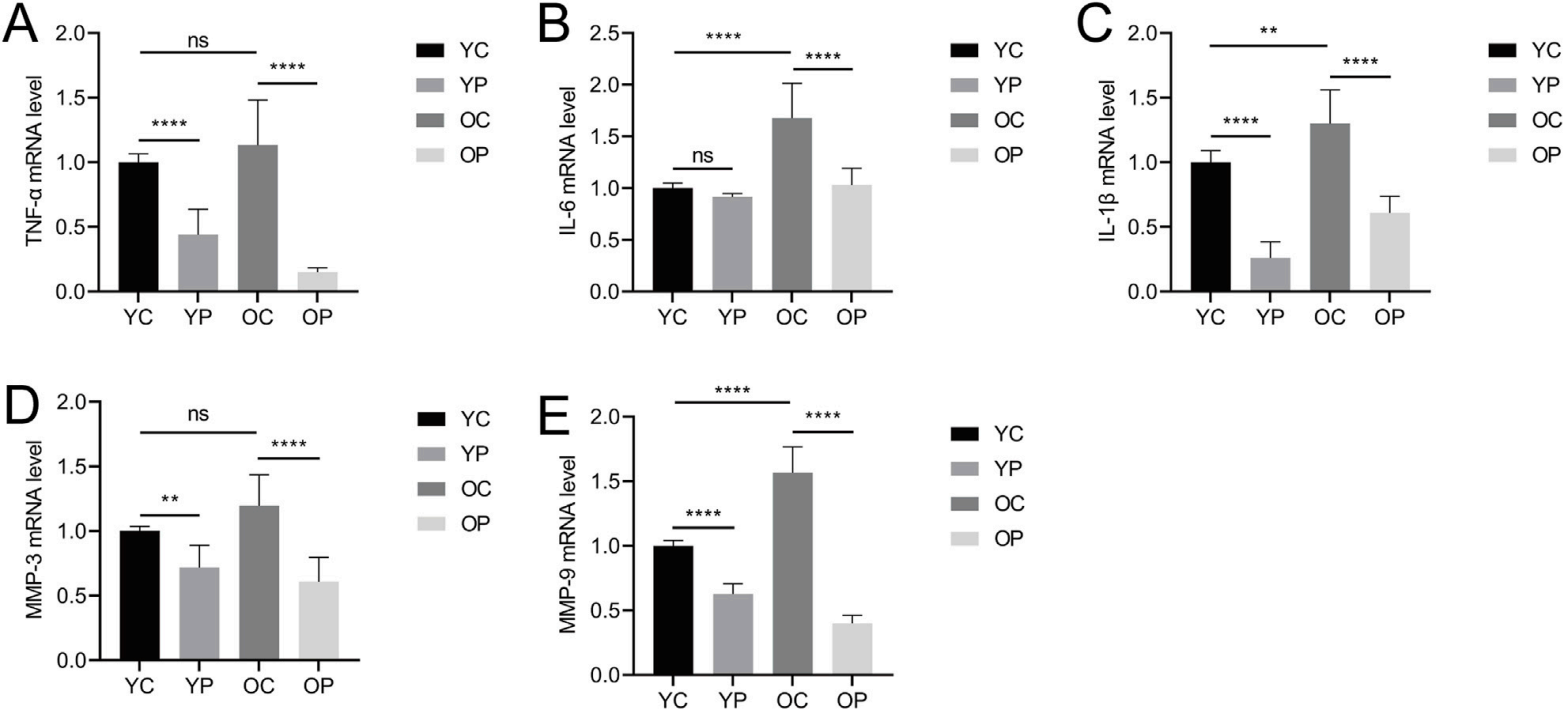
The Effect of LTCP on the Expression of Inflammatory Factors and Senescence-Associated Secretory Phenotype Factors. **(A)** mRNA expression level of *TNF-α*. **(B)** mRNA expression level of *IL-6*. **(C)** mRNA expression level of *IL-1β*. **(D)** mRNA expression level of *MMP-3*. **(E)** mRNA expression level of *MMP-9*. Data are presented as mean ± SD (n = 3). *P < 0.05, **P < 0.01, and ****P < 0.0001. P-values were calculated using One-Way ANOVA.

Delayed wound healing can be attributed to a widespread inflammatory response primarily driven by bacterial infection, leading to failed macrophage polarization and excessive secretion of inflammatory factors such as TNF-α, IL-6, and IL-1β (Zhang et al., 2023). As shown in Figures 4A–C, the mRNA expression levels of *TNF-α*, *IL-6*, and *IL-1β* in the YP and OP groups were lower compared to the YC and OC groups. Specifically, the mRNA expression levels of *TNF-α* and *IL-1β* in the YP group were significantly downregulated by 55.98% and 73.91%, respectively (P < 0.05), while the *IL-6* mRNA expression level in the YP group decreased by 8.44% (P > 0.05). Compared with the OC group, the mRNA expression levels of *TNF-α*, *IL-6*, and *IL-1β* in the OP group were significantly reduced by 86.61%, 38.65%, and 53.11%, respectively (P < 0.05). In contrast, the mRNA expression levels of *TNF-α*, *IL-6*, and *IL-1β* were elevated in the OC group compared to the YC group, indicating that the post-injury inflammatory response was more pronounced in aged mice. These results suggested that LTCP could inhibit the expression of inflammatory factors and reduce the levels of local inflammatory responses, thereby promoting wound healing.

Senescent cells accumulate during the aging process, promoting chronic inflammation, altering the tissue microenvironment, and modifying the function of adjacent cells, these physiological changes are associated with the senescence-associated secretory phenotype (SASP) (Kim D. E. et al., 2020). In this study, we detected the expression levels of matrix metalloproteinase MMP-3 and MMP-9, which are SASP factors. As illustrated in Figures 4D,E, the mRNA expression levels of *MMP-3* and *MMP-9* in the YP and OP groups were significantly downregulated compared to the YC and OC groups (P < 0.05). Specifically, the mRNA expression levels of *MMP-3* and *MMP-9* in the YP group decreased by 28.15% and 37.16%, respectively (P < 0.05). In comparison to the OC group, the mRNA expression levels of *MMP-3* and *MMP-9* in the OP group were reduced by 49.17% and 74.50%, respectively (P < 0.05). Additionally, the mRNA expression levels of *MMP-3* and *MMP-9* were elevated in the OC group relative to the YC group, consistent with the expression levels of *TNF-α*, *IL-6*, and *IL-1β*, indicating the presence of an age-related inflammatory phenotype. These results suggested that LTCP could effectively modulate the expression levels of SASP factors, thereby improving the delayed wound healing associated with aging.

### 3.3 The effect of LTCP on the expression of tissue repair-related factors

**FIGURE 5:**
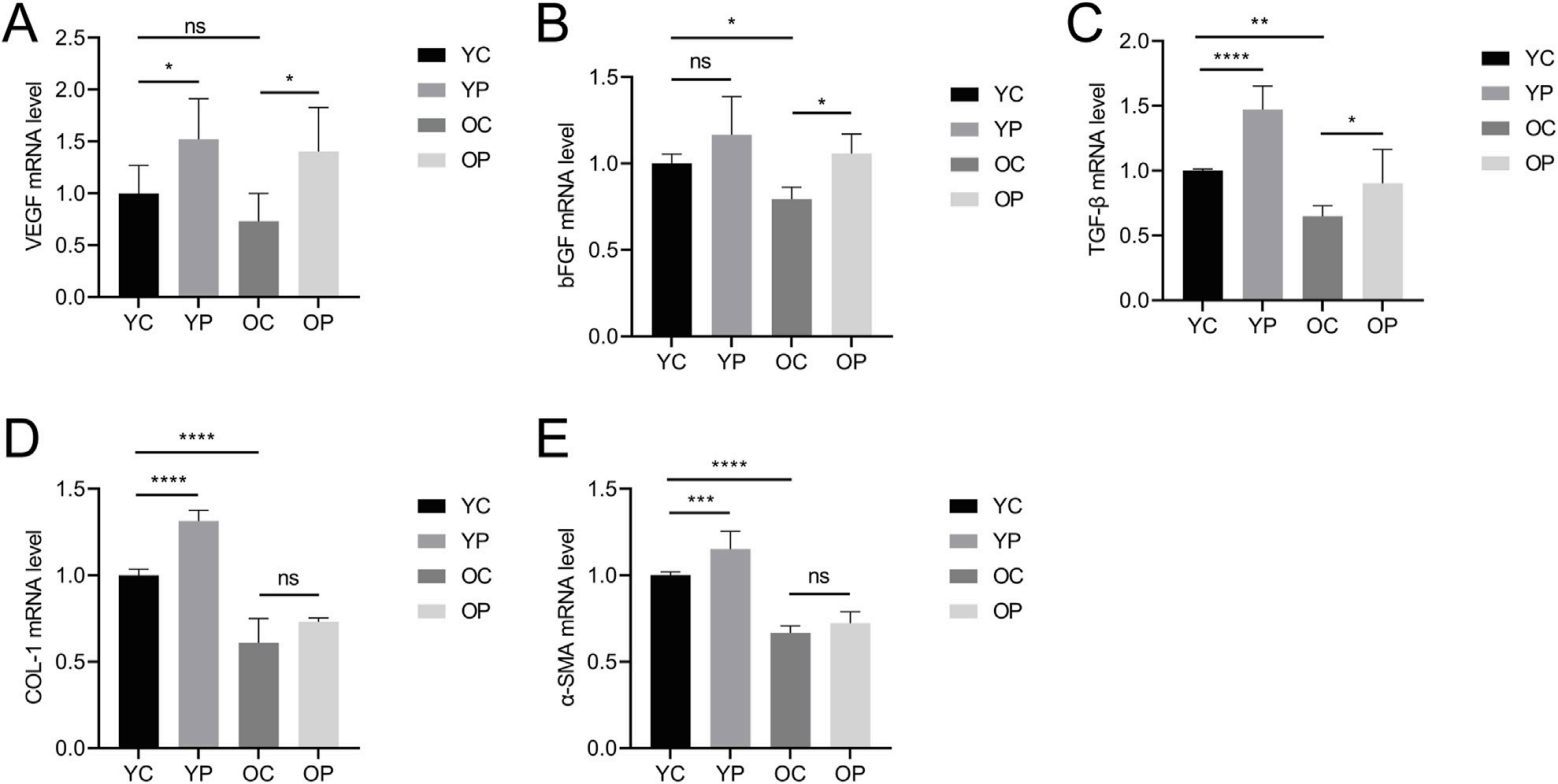
The Effect of LTCP on the Expression of Tissue Repair-Related Factors. **(A)** mRNA expression level of *VEGF*. **(B)** mRNA expression level of *bFGF*. **(C)** mRNA expression level of *TGF-β*. **(D)** mRNA expression level of *COL-I*. **(E)** mRNA expression level of *α-SMA*. Data are presented as mean ± SD (n = 3). *P < 0.05, **P < 0.01, ***P < 0.001, ****P < 0.0001. P-values were calculated using One-Way ANOVA.

The wound healing process is highly regulated by the secretion of various growth factors, cytokines, and chemokines (Nourian Dehkordi et al., 2019). In this study, we measured the expression levels of vascular endothelial growth factor (VEGF), basic fibroblast growth factor (bFGF), transforming growth factor-β (TGF-β), type I collagen (COL-I), and α-smooth muscle actin (α-SMA) using qRT-PCR. As shown in Figures 5A–E, the mRNA expression levels of *VEGF*, *bFGF*, *TGF-β*, *COL-I*, and *α-SMA* were significantly increased in the YP and OP groups compared to the YC and OC groups. Specifically, compared with the YC group, the mRNA expression levels of *VEGF*, *TGF-β*, *COL-I*, and *α-SMA* in the YP group were significantly upregulated by 52.26%, 47.04%, 31.45%, and 15.21%, respectively (P < 0.05). The mRNA expression level of *bFGF* in the YP group was upregulated by 16.73% (P > 0.05). Compared with the OC group, the mRNA expression levels of *VEGF*, *bFGF*, and *TGF-β* in the OP group were significantly upregulated by 91.59%, 33.38%, and 39.48%, respectively (P < 0.05), while the mRNA expression levels of *COL-I* and *α-SMA* increased by 19.86% and 8.51%, respectively (P > 0.05). In contrast, the mRNA expression levels of *VEGF*, *bFGF*, *TGF-β*, *COL-I*, and *α-SMA* in the OC group showed varying degrees of decline compared to the YC group, indicating a diminished regenerative repair capacity in aged skin tissue. These results suggested that LTCP could promote the expression of tissue repair-related factors, thereby accelerating wound healing.

### 3.4 The effect of LTCP on epidermal cell proliferation and apoptosis

**FIGURE 6:**
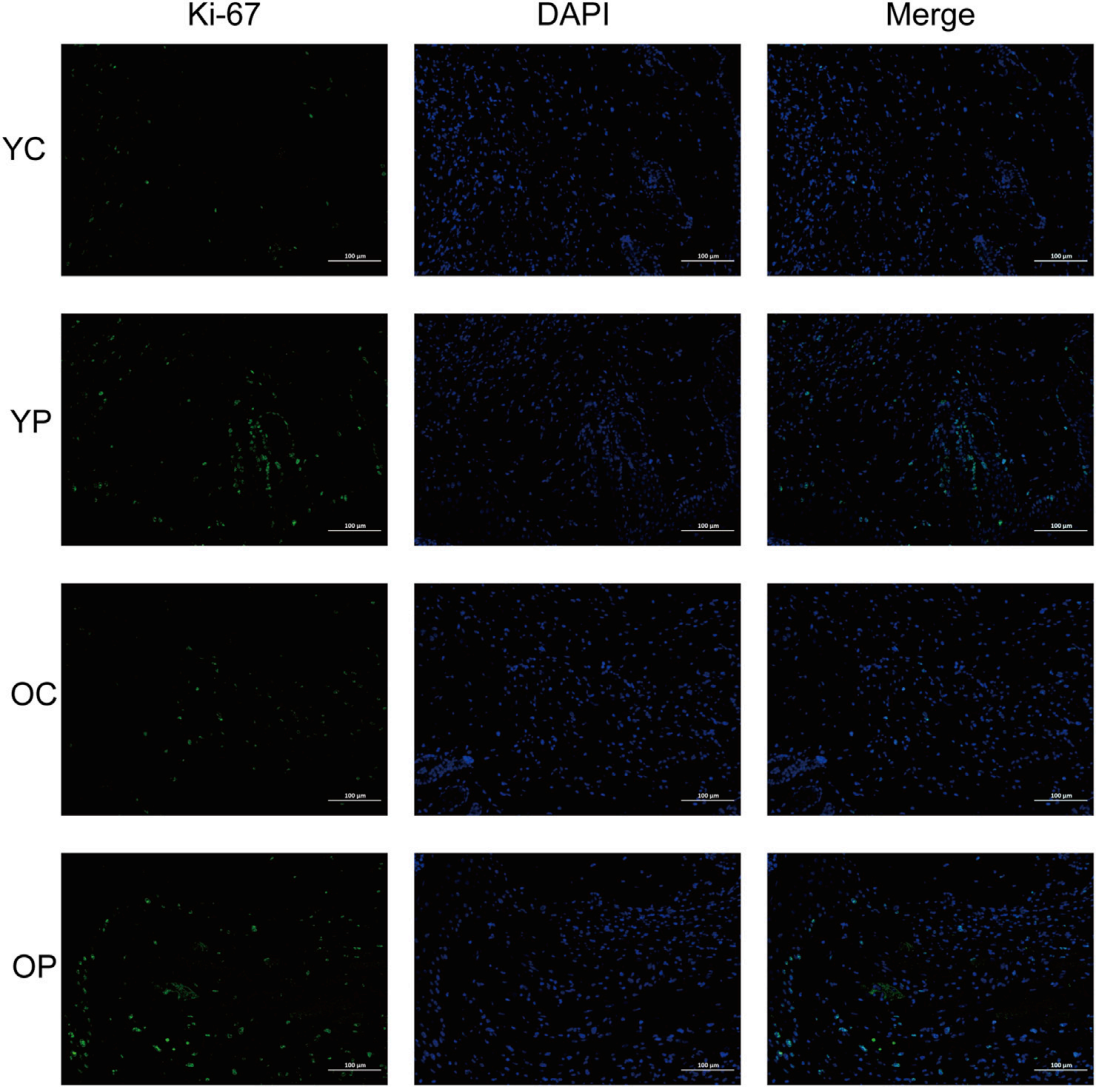
The Effect of LTCP on Epidermal Cell Proliferation. Immunofluorescent staining analysis of the cell proliferation marker Ki-67 on day 3 post-surgery across all groups. n = 3, scale bar = 100 μm.

**FIGURE 7:**
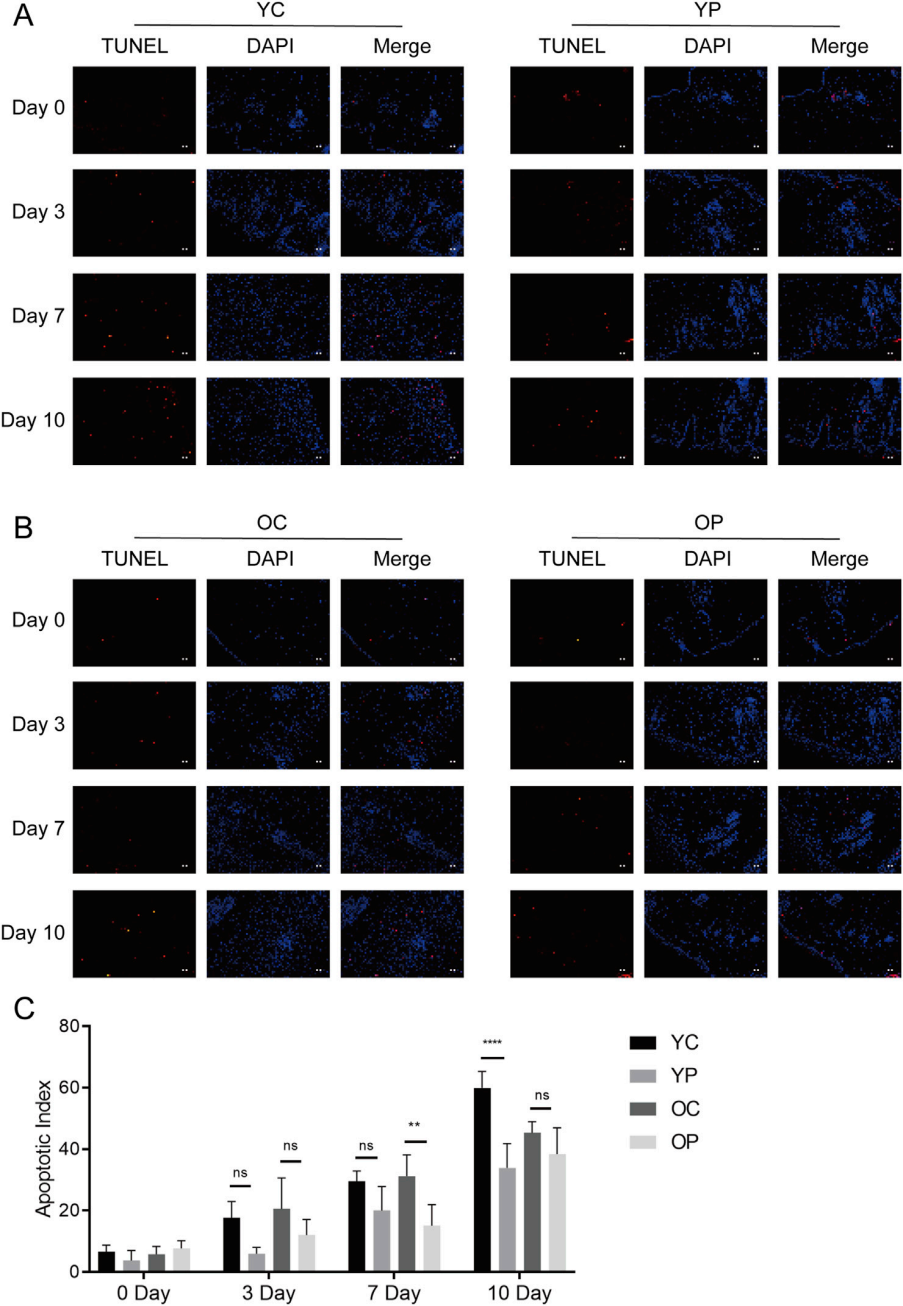
The Effect of LTCP on Epidermal Cell Apoptosis. **(A)** TUNEL staining analysis of the YC and YP groups on days 0, 3, 7, and 10 post-surgeries. **(B)** TUNEL staining analysis of the OC and OP groups on days 0, 3, 7, and 10 post-surgeries. n = 3, scale bar = 100 μm. **(C)** The Apoptotic Index on days 0, 3, 7, and 10 post-surgeries across all groups. Data are presented as mean ± SD (n = 3). **P < 0.01, ****P < 0.0001. P-values were calculated using Two-Way ANOVA.

Proliferation and apoptosis are prerequisites for wound healing (Liao et al., 2020). The assessment of cell proliferation and apoptosis during the skin wound healing process was conducted using immunofluorescent staining for the cell proliferation marker Ki-67 and TUNEL staining. As illustrated in Figure 6, on day 3 post-surgery, there was a marked increase in cell proliferation in the wound areas of the YP and OP groups compared to the YC and OC groups. Furthermore, as shown in Figures 7A–C, the number of apoptotic cells significantly increased across all groups on days 3, 7, and 10 post-surgeries. However, compared with the YC and OC groups, the YP and OP groups exhibited a notable reduction in the number of apoptotic cells on days 3, 7, and 10 post-surgeries. These results indicated that LTCP could inhibit apoptosis and promote cell proliferation, thereby maintaining the homeostatic balance of cell proliferation and apoptosis during the wound healing process.

### 3.5 The effect of LTCP on genes expression in skin wound tissue

**FIGURE 8:**
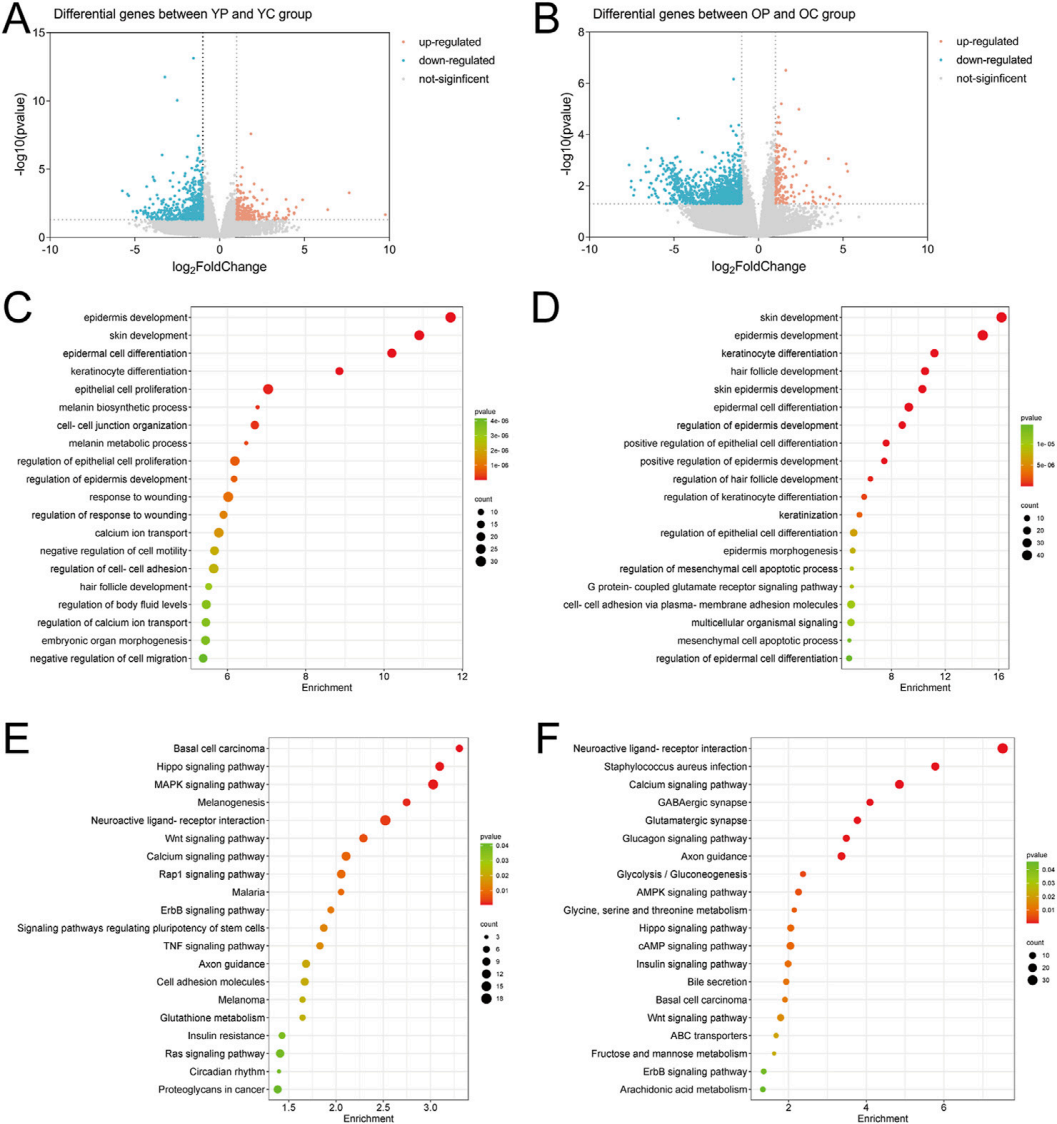
The Effect of LTCP on Genes Expression in Skin Wound Tissue. **(A)** Volcano plot of DEGs in the YP vs. YC comparison group. **(B)** Volcano plot of DEGs in the OP vs. OC comparison group. **(C)** GO analysis of DEGs in the YP vs. YC comparison group. **(D)** GO analysis of DEGs in the OP vs. OC comparison group. **(E)** KEGG analysis of DEGs in the YP vs. YC comparison group. **(F)** KEGG analysis of DEGs in the OP vs. OC comparison group. n = 3.

On the third postoperative day, skin tissue surrounding the wounds from each experimental group was collected for transcriptomic analysis and differentially expressed genes (DEGs) analysis using p-value <0.05 and | Log2FoldChange | ≥ 1 as screening criteria. Compared with the YC group, the YP group exhibited a total of 191 significantly upregulated DEGs and 527 significantly downregulated DEGs; the OP group compared to the OC group showed 155 significantly upregulated DEGs and 1,104 significantly downregulated DEGs (Figures 8A,B). To explore the potential biological functions of these DEGs, we performed functional analysis using the Cluster Profiler database. GO enrichment analysis showed that the DEGs obtained from the YP group compared to the YC group were primarily involved in biological processes such as skin and epidermal development, differentiation of epidermal and keratinocytes, epithelial cell proliferation, cell-cell adhesion, and regulation of body fluid levels (Figure 8C). The DEGs obtained from the OP group compared to the OC group primarily participated in skin and epidermal development, differentiation of epidermal and keratinocytes, epithelial cell differentiation, mesenchymal cell apoptosis, and cell-cell adhesion (Figure 8E). KEGG pathway analysis revealed that the DEGs from the YP group compared to the YC group were mainly enriched in the Hippo, MAPK, and Wnt signaling pathways (Figure 8D), while the DEGs from the OP group compared to the OC group were primarily enriched in pathways related to *Staphylococcus aureus* infection, Hippo, and Wnt signaling (Figure 8F).

**FIGURE 9:**
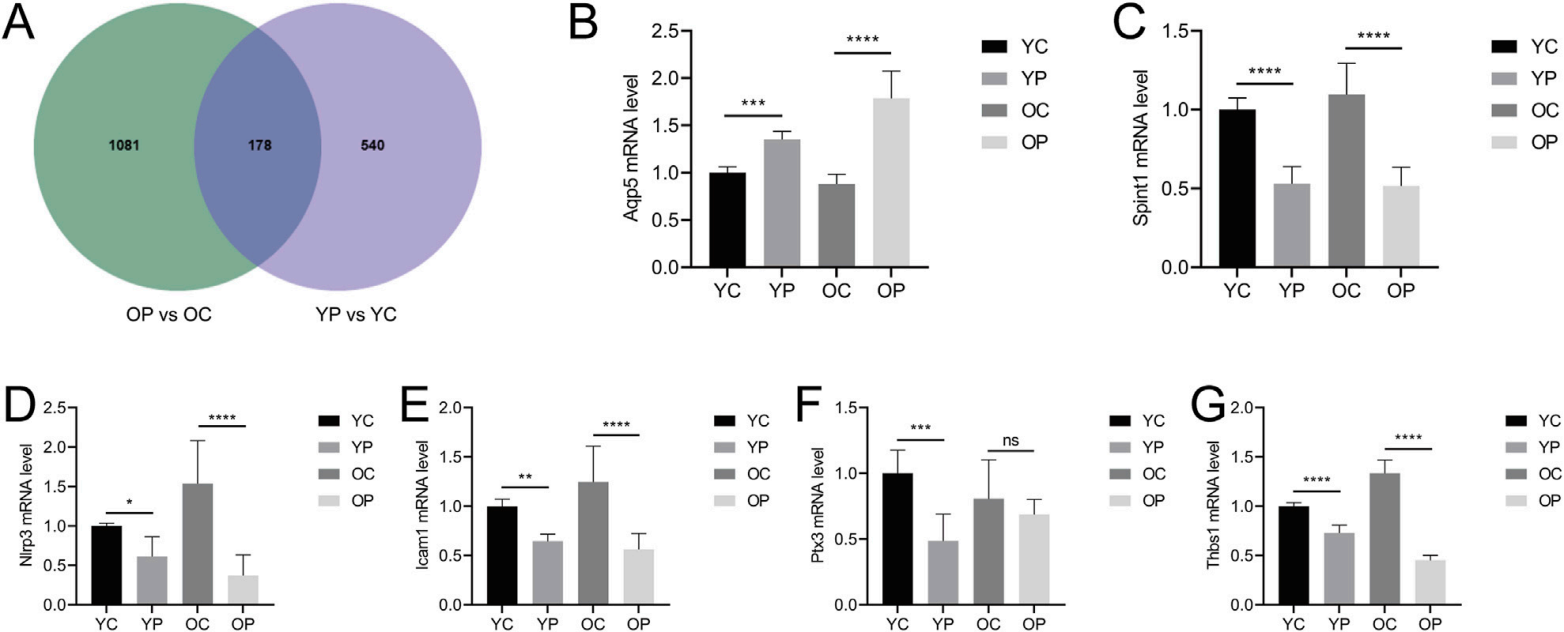
Venn analysis of differentially expressed genes and the effects of LTCP on gene expression related to inflammatory response, cell proliferation, migration, and angiogenesis. **(A)** Venn diagram of DEGs in the YP vs. YC and OP vs. OC comparison groups. **(B)** mRNA expression level of *Aqp5* **(C)** mRNA expression level of *Spint1*. **(D)** mRNA expression level of *Nlrp3*. **(E)** mRNA expression level of *Icam1*. **(F)** mRNA expression level of *Ptx3*. **(G)** mRNA expression level of *Thbs1*. Data were presented as mean ± SD (n = 3). *P < 0.05, **P < 0.01, ***P < 0.001, ****P < 0.0001. P-values were calculated using One-Way ANOVA.

The Venn diagram of DEGs for the two comparisons groups, YP vs. YC and OP vs. OC, showed a total of 178 overlapping genes (Figure 9A). Six genes related to inflammatory response, cell proliferation and migration, and angiogenesis were selected for mRNA level validation by qRT-PCR. As shown in Figures 9B–G, compared with the YC and OC groups, the mRNA expression level of *Aqp5* in the YP and OP groups was significantly upregulated (P < 0.05), while the mRNA expression levels of *Spint1*, *Nlrp3*, *Icam1*, *Ptx3*, and *Thbs1* were significantly downregulated (P < 0.05). These results indicated that LTCP could effectively modulate the expression levels of genes associated with cell proliferation and migration (*Aqp5*, *Spint1*), inflammatory response (*Nlrp3*, *Icam1*), and angiogenesis (*Ptx3*, *Thbs1*), thereby promoting wound healing.

### 3.6 The effect of LTCP on skin microbiome

The skin serves as a habitat for various pathogenic and symbiotic bacteria. While these bacteria maintain a balanced state in healthy skin, skin injury can disrupt this equilibrium, leading to delayed wound healing (Ersanli et al., 2023). To explore the impact of LTCP on the skin microbiome, 16S rRNA high-throughput sequencing analysis of the microbial composition in skin wounds was conducted. Adult and aged mice, were sampled before wounding, immediately after wounding, and 2 min after treatment with LTCP, and divided into six experimental groups: Old Control (OC), Old +0 min (O0), Old +2 min (O2), Young Control (YC), Young +0 min (Y0), and Young +2 min (Y2).

**FIGURE 10:**
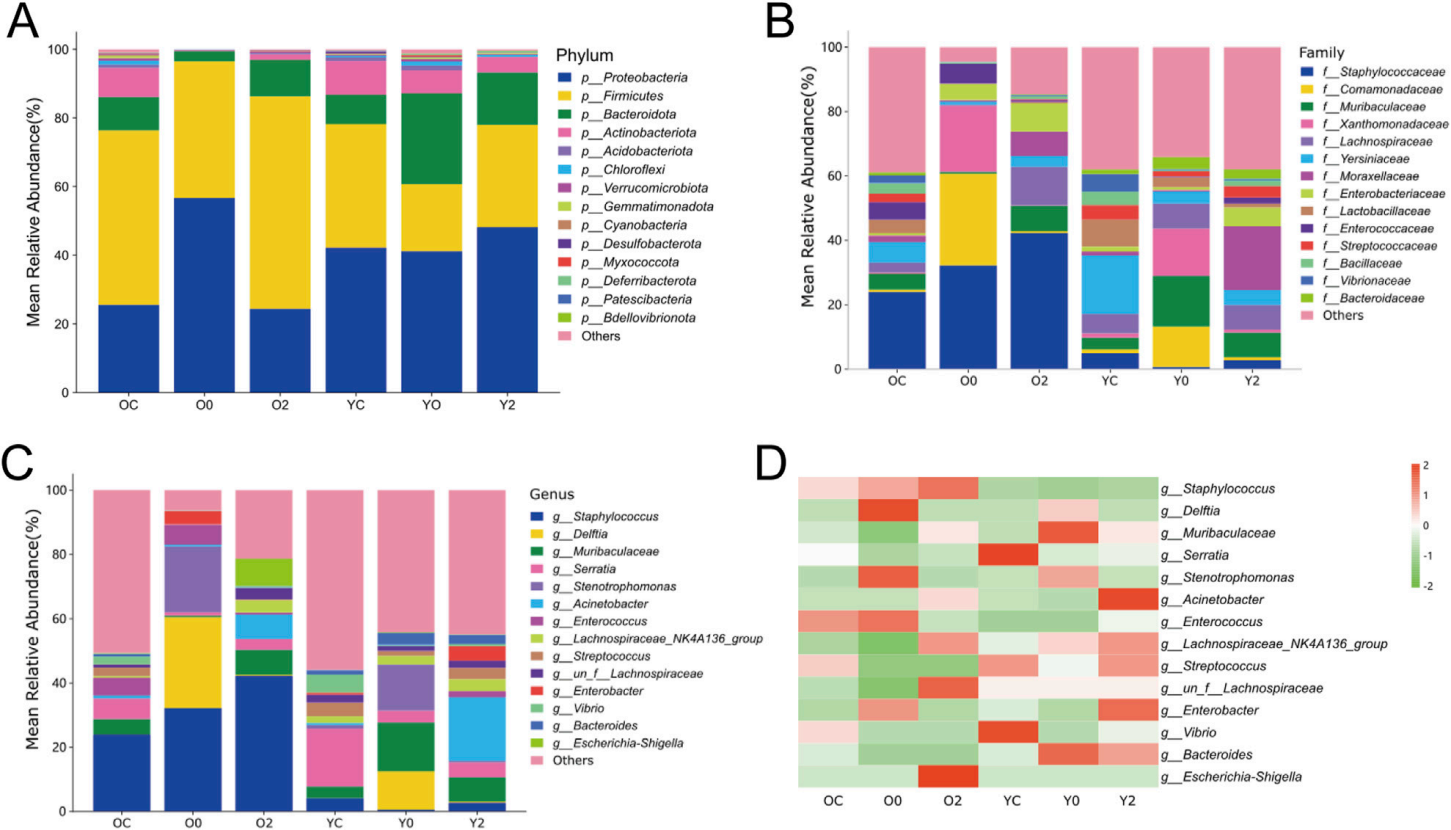
The Effect of LTCP on Skin Microbiome. **(A)** Bar chart of relative abundance of species at the phylum level. **(B)** Bar chart of relative abundance of species at the family level. **(C)** Bar chart of relative abundance of species at the genus level. **(D)** Heatmap of species abundance at the genus level.

The differences in species composition and relative abundance of the skin wound microbiome across the groups were analyzed. The bar chart of relative abundance of species at the phylum level (Figure 10A) indicated that *Proteobacteria*, *Firmicutes*, *Bacteroidota*, and *Actinobacteriota* were the dominant phyla in the microbial community of mouse skin wounds, with *Proteobacteria* and *Firmicutes* being the most prevalent. The average abundance of *Proteobacteria* in the three treatment groups of aged mice was 25.56%, 56.65%, and 24.40%, respectively, and in the young mice’s three treatment groups, it was 42.17%, 41.16%, and 48.13%. The average abundance of *Firmicutes* in the three treatment groups of aged mice was 50.82%, 39.86%, and 61.93%, and in the young mice’s groups, it was 36.03%, 19.56%, and 29.88%. These results suggested that LTCP could reduce the relative abundance of *Proteobacteria* and increase the relative abundance of *Firmicutes*, thereby improving the microbial community structure in skin wounds. The composition of the skin wound microbiome at the family level was shown in Figure 10B. The families with an average relative abundance greater than 1% across all groups included *Staphylococcaceae*, *Comamonadaceae*, *Muribaculaceae*, *Xanthomonadaceae*, *Lachnospiraceae*, *Yersiniaceae*, *Moraxellaceae*, *Enterobacteriaceae*, *Lactobacillaceae*, *Enterococcaceae*, *Streptococcaceae*, *Bacillaceae*, *Vibrionaceae*, and *Bacteroidaceae*. The relative abundance and differences of the skin wound microbiome at the genus level were presented in Figures 10C,D. Compared with the OC group, the O0 group exhibited an increased relative abundance of *Delftia*, *Stenotrophomonas*, *Enterococcus*, and *Enterobacter*, while the relative abundance of *Muribaculaceae*, *Acinetobacter*, *Lachnospiraceae_NK4A136_group*, and *un_f__Lachnospiraceae* decreased. The relative abundance of *Delftia*, *Stenotrophomonas*, *Enterococcus*, and *Enterobacter* of the O2 group decreased compared to the O0 group, while *Muribaculaceae*, *Acinetobacter*, *Lachnospiraceae_NK4A136_group*, and *un_f__Lachnospiraceae* showed varying degrees of increase. Compared with the YC group, the Y0 group showed an increase in the relative abundance of *Delftia* and *Stenotrophomonas*, while *Acinetobacter* and *un_f__Lachnospiraceae* decreased. The relative abundance of *Acinetobacter* and *un_f__Lachnospiraceae* of the Y2 group increased compared to the Y0 group, while *Delftia* and *Stenotrophomonas* showed a decrease. These results suggested that LTCP could effectively reduce harmful bacteria such as *Delftia*, *Stenotrophomonas*, *Enterococcus*, and *Enterobacter* in skin wounds, while increasing beneficial bacteria levels such as *Muribaculaceae*, *Acinetobacter*, *Lachnospiraceae_NK4A136_group*, and *un_f__Lachnospiraceae*, thereby positively modulating the skin microbiome and promoting the skin repair process.

## 4 Discussion

In recent years, due to the global aging population and the rising prevalence of chronic diseases, the incidence rate of chronic wounds has gradually increased (Manchanda et al., 2023). Finding effective methods for chronic wound treatment has become a focal point of clinical research. Current therapeutic approaches for skin wound repair include medications, wound dressings, growth factors, electrical stimulation, negative pressure, and hyperbaric oxygen (Yu et al., 2022). However, these treatment methods have certain limitations concerning drug resistance, healing rates, ease of application, and treatment cycle.

Low-temperature plasma is widely utilized in various biomedical fields, including sterilization, hemostasis, oral treatment, skin wound healing, and tumor therapy (Wu et al., 2023). The dielectric barrier discharge plasma device consists of two flat metal electrodes covered with dielectric materials, with human tissue acting as the counter electrode (Dubey et al., 2022). Direct discharge devices such as dielectric barrier discharge can more easily control the plasma composition, however, they require maintaining an effective distance between the electrode and tissue, typically less than 3 mm, which limits their application in small areas of the human body (Dubuc et al., 2018). The indirect discharge plasma devices do not use the target area as a counter electrode. Plasma is generated between two electrodes within the device and delivered to the target area via carrier gas or diffusion (Isbary et al., 2013). By adjusting the type of gas source to change the active substance composition of the plasma, thereby obtaining plasma suitable for specific applications (Reuter et al., 2018). However, these devices necessitate additional gas supply equipment, have a limited action area, and are not portable (Isbary et al., 2013). Various factors, including gas composition, power, pressure, and frequency used to perform operations, often influence the concentration and properties of the chemicals produced in the plasma (Ishaq et al., 2015). Studies have shown that treating tissue with LTCP for 1–3 min can significantly enhance the proliferation of keratinocytes, which are beneficial for epidermal regeneration during the wound healing process (Hasse et al., 2016). Najafzadehvarzi et al. used LTCP direct irradiation to treat healthy rat skin tissue and found that the direct treatment time was safe within 2–5 min without any toxic side effects (Najafzadehvarzi et al., 2022). However, this study only conducted a single treatment with LTCP, lacking observations of long-term cumulative effects, and there was not further examination of the pathological microstructure of the local skin tissue. We developed a LTCP device based on a single-electrode dielectric barrier air discharge mode. Compared to devices generating plasma discharge directly driven by a 220 V power supply, its discharge produces stronger electromagnetic radiation to the external environment. Plasma discharge connected to a 220 V power supply typically uses DC or low-frequency AC voltage to ionize gas, generating plasma. In contrast, dielectric barrier low-temperature plasma discharge employs a high-frequency AC voltage with an insulating dielectric between electrodes, enabling more uniform, stable plasma at lower voltages (Isbary et al., 2013; Dubey et al., 2022). While 220 V plasma discharge radiation mainly originates from electron-ion interactions and environmental effects, LTCP devices produce stronger electromagnetic radiation due to additional charge accumulation, polarization, and micro-discharge dynamics caused by the dielectric and high-frequency voltage (Kogelschatz, 2003). In this study, we established skin injury models on the backs of mice of different ages and treated them with a self-developed LTCP device, recording and analyzing wound images and areas from postoperative days 0–12. The results showed that, compared with the YC and OC groups, LTCP treatment accelerated wound healing in the YP and OP groups. Morphological observations of the skin wound tissue in mice indicated that LTCP reduced the infiltration of inflammatory cells in tissues and promoted the formation of granulation tissue.

Wound healing involves numerous coordinated biological processes, including inflammation, and persistent inflammatory responses can lead to delayed healing (Zhang et al., 2021), characterized by increased expression levels of pro-inflammatory cytokines such as TNF-α, IL-6, and IL-1β (Eming et al., 2014; Sun et al., 2021), which is consistent with the results of this study. The skin wounds in the OC group of mice exhibited higher levels of *TNF-α*, *IL-6*, and *IL-1β* compared to the YC group. Additionally, it was found that LTCP could reduce the expression levels of *TNF-α*, *IL-6*, and *IL-1β* in wounds of mice of different ages, thereby improving the inflammatory response and accelerating the wound healing process (Figures 4A–C).

Aging is also one of the contributing factors to chronic wounds (Las Heras et al., 2020). The significant features of skin aging in older adults are the thinning of the epidermis and dermis, accompanied by loss of moisture and collagen, leading to increased skin fragility, impaired vascular support, delayed wound healing, and heightened susceptibility to cancer development (Quan, 2023). A major hallmark of aging is the chronic accumulation of senescent cells, which release chemokines, inflammatory cytokines, and proteases, negatively impacting the tissue microenvironment, a phenomenon known as the Senescence-Associated Secretory Phenotype (SASP) (Cai et al., 2020; Dungan et al., 2020). Elevated expression levels of matrix metalloproteinases (MMP-3/9), markers of SASP (Bulbiankova et al., 2023), cause excessive degradation of collagen and ECM, hindering re-epithelialization and reducing the efficiency of cell proliferation and migration, ultimately resulting in delayed wound healing (Nguyen et al., 2016). In this study, it was observed that post-injury skin tissues expressed high levels of *MMP-3* and *MMP-9*, with even higher expression levels in the OC group. Conversely, after treatment with LTCP, the YP and OP groups exhibited significantly decreased expression levels of *MMP-3* and *MMP-9*, indicating that LTCP can reduce the expression of SASP factors and improve wound healing delays in aged skin (Figures 4D,E).

Cell proliferation, migration, angiogenesis, granulation tissue formation, collagen deposition, re-epithelialization, and wound contraction are all critical process for wound healing (Rezaie et al., 2019). Key regulatory factors involved in skin wound healing include VEGF, bFGF, TGF-β, COL-I, and α-SMA (Gonzalez et al., 2016). Previous studies have found that aging skin exhibits decreased levels of growth factors such as VEGF, bFGF, and TGF-β, along with reduced collagen content and differentiation of myofibroblasts when compared to younger skin (Fujiwara et al., 2016; Bonham et al., 2020; Liu et al., 2022). Our results are consistent with these findings, the expression levels of *VEGF*, *bFGF*, *TGF-β*, *COL-I* and *α-SMA* in the skin wounds of the OC group were lower compared to those in the YC group. LTCP treatment significantly increased the expression levels of these tissue repair-related factors (Figures 5A–E). Additionally, the repair of skin damage involves the proliferation, differentiation, migration, and apoptosis of various cell types (Subramaniam et al., 2021). Research has shown that LTCP can enhance wound healing by promoting angiogenesis, cell proliferation, migration, and resistance to apoptosis (Ma et al., 2023), our study also corroborates these findings (Figures 6, 7). Meanwhile, previous studies indicated that aquaporin 5 (Aqp5) plays a role in regulating the proliferation and differentiation of epidermal keratinocytes (Zhou et al., 2020); Serine Peptidase Inhibitor, Kunitz Type 1 (Spint1), a serine protease inhibitor, can bind to hepatocyte growth factor activator (HGFA), thereby blocking the activation of HGF and plays a role in the skin and intestines (Conway et al., 2007; Kataoka et al., 2018); NLR Family Pyrin Domain Containing 3 (Nlrp3) is crucial for macrophage regulation of IL-1β in the inflammatory response and is closely related to the activation and sustained inflammatory response in wounds and macrophage phenotype (Weinheimer-Haus et al., 2015). Intercellular adhesion molecule-1 (Icam1), a membrane-bound glycoprotein, its upregulation is a hallmark event during inflammation (Ramos et al., 2014); Pentraxin 3 (Ptx3) is a key activator of inflammatory and repair factors following tissue injury and can inhibit FGF-mediated angiogenesis and the proliferation of smooth muscle cells (Cappuzzello et al., 2016; Presta et al., 2018); Thrombospondin-1 (Thbs1) inhibits the migration and proliferation of endothelial cells by regulating the CD36 and CD47 receptors, promoting endothelial cell apoptosis and thereby suppressing angiogenesis; downregulation of Thbs1 may facilitate wound healing (Bi et al., 2019). Our study found that LTCP significantly increased the expression of *Aqp5* while inhibiting *Spint1*, *Nlrp3*, *Icam1*, *Ptx3*, and *Thbs1*, thereby promoting cell proliferation and migration, alleviating the inflammatory response, increasing angiogenesis, and facilitating wound healing. Additionally, the study on other DEGs after LTCP treatment revealed that DEGs were primarily enriched in pathways related to *Staphylococcus aureus* infection and the Hippo, MAPK, and Wnt signaling pathways. *Staphylococcus aureus* is one of the most common and significant pathogens, often leading to persistent wound infections and adverse reactions. Moreover, biofilm infections caused by pathogenic bacteria, including *Staphylococcus aureus*, are a major reason for delayed wound healing, with an approximate prevalence of 78.2% of biofilm infections in chronic wounds (Roy et al., 2020; Ersanli et al., 2023). Previous studies have demonstrated that LTCP can effectively eliminate *Staphylococcus aureus* and the Hippo, MAPK, and Wnt signaling pathways play crucial roles in various processes of wound healing, corroborating our findings (Duarte and Panariello, 2020; Zulkefli et al., 2023).

Wounds provide an opportunity for skin surface microbes that constitute the skin microbiome, as well as microorganisms in the environment, to enter the deeper tissues and find optimal conditions for colonization and growth (Tomic-Canic et al., 2020). Pathogenic bacteria can lead to infections at the wound site, resulting in delayed or impaired healing. Conversely, probiotics can favorably affect wound healing by providing a barrier function to the skin and combating pathogenic microorganisms (Ersanli et al., 2023). Research has shown that LTCP can effectively kill various types of bacteria, including Gram-positive and Gram-negative bacteria, anaerobes, aerobes, and facultative anaerobes (Bolgeo et al., 2023). However, current research on the bactericidal effect of LTCP has only been conducted through the treatment of pathogenic bacteria in infected tissues or cells, and systematic studies on beneficial bacteria and skin wound microbiota have not been reported. Our results revealed that the dominant phyla in the skin wounds of mice were *Proteobacteria*, *Firmicutes*, *Bacteroidota*, and *Actinobacteriota*, with *Proteobacteria* and *Firmicutes* being the most prevalent (Figure 10A). After skin injury, the relative abundance of *Proteobacteria*, *Delftia*, *Stenotrophomonas*, *Enterococcus*, and *Enterobacter* increased, while the relative abundance of *Firmicutes*, *Muribaculaceae*, *Acinetobacter*, *Lachnospiraceae_NK4A136_group*, and *un_f__Lachnospiraceae* decreased (Figures 10C,D). LTCP treatment effectively improved these shifts in relative abundance of these bacteria, reducing harmful bacteria while increasing beneficial ones, thereby restoring skin microbiota homeostasis. *Delftia*, an environmental bacterium, can cause severe infections, such as pneumonia, sepsis, and endocarditis in immunocompetent hosts, and is highly associated with the microenvironment of skin wounds, exhibiting resistance to β-lactam antibiotics (Sohn and Baek, 2015; Ranc et al., 2018; Kim J. H. et al., 2020; Ryan et al., 2022). *Stenotrophomonas* can secrete LPS, inducing the production of pro-inflammatory factors such as NO, IL-6, and TNF-α while inhibiting IL-10 secretion, thereby promoting inflammatory responses that affect tissue repair (Mei et al., 2020). *Enterococcus* and *Enterobacter* are common bacteria responsible for postoperative wound infections as well as skin and soft tissue infections (Wang C.-H. et al., 2021). Mi et al. found that *Enterococcus* was associated with the exacerbation of diabetic wound infections (Mi et al., 2022). However, other studies reported that *Enterococcus* might play a positive role in wound healing (Mei et al., 2020). An *in vivo* and *in vitro* animal study indicated that *Acinetobacter* could induce strong T_H_1 and anti-inflammatory responses via immune and skin cells, preventing skin allergic inflammation and lung inflammation (Fyhrquist et al., 2014). *Muribaculaceae* and *Lachnospiraceae* are significant metabolically active groups in the skin, with *Muribaculaceae* contributing to propionate production and *Lachnospiraceae* playing an essential role in butyrate production (Sibai et al., 2020). Notably, the relative abundance of *Muribaculaceae* increased in the injured skin of older mice and significantly decreased following LTCP treatment, whereas the trend in younger mice was the opposite. Therefore, we hypothesize that changes in the relative abundance and function of *Muribaculaceae* may be age-related.

Our study demonstrates that LTCP can reduce the infiltration of inflammatory cells in injured tissues, promote granulation tissue formation, downregulate the expression of various pro-inflammatory factors and SASP factors, and maintain the balance between cell proliferation and apoptosis in tissue. Furthermore, it effectively modulates the expression levels of multiple tissue repair-related factors, and stabilizes the skin microbiota by reducing harmful bacteria and increasing beneficial ones. This provides experimental evidence for the clinical application of LTCP in promoting skin wound healing in mice of different ages, especially in aging mice. At the same time, LTCP has a profound impact on the structure and abundance of microbial communities in skin wounds, which has been overlooked in previous studies. This contributes to the understanding of the mechanism of skin wound occurrence and the development of microbial agents for skin wound healing.

## Data Availability

The datasets presented in this study can be found in online repositories. The names of the repository/repositories and accession number(s) can be found below: https://www.ncbi.nlm.nih.gov/, PRJNA1169326.

## References

[B1] Archer N. K. Wang Y. Ortines R. V. Liu H. Y. Nolan S. J. Liu Q. (2020). “Preclinical models and methodologies for monitoring *Staphylococcus aureus* infections using noninvasive optical imaging,” in Methicillin-resistant Staphylococcus aureus (MRSA) protocols. Editor Ji Y. (New York, NY: Springer US), 197–228. 10.1007/978-1-4939-9849-4_15 PMC7745539 31523776

[B2] Bi H. S. Li H. Zhang C. Mao Y. Q. Nie F. F. Xing Y. (2019). Stromal vascular fraction promotes migration of fibroblasts and angiogenesis through regulation of extracellular matrix in the skin wound healing process. Stem Cell Res. Ther. 10, 302. 10.1186/s13287-019-1415-6 31623669 PMC6798485

[B3] Boekema B. Stoop M. Vlig M. Van Liempt J. Sobota A. Ulrich M. (2021). Antibacterial and safety tests of a flexible cold atmospheric plasma device for the stimulation of wound healing. Appl. Microbiol. Biotechnol. 105, 2057–2070. 10.1007/s00253-021-11166-5 33587156 PMC7906937

[B4] Bolgeo T. Maconi A. Gardalini M. Gatti D. Di Matteo R. Lapidari M. (2023). The role of cold atmospheric plasma in wound healing processes in critically ill patients. J. Pers. Med. 13, 736. 10.3390/jpm13050736 37240907 PMC10219374

[B5] Bonham C. A. Kuehlmann B. Gurtner G. C. (2020). Impaired neovascularization in aging. Adv. Wound Care 9, 111–126. 10.1089/wound.2018.0912 PMC6985771 31993253

[B6] Bulbiankova D. Díaz-Puertas R. Álvarez-Martínez F. J. Herranz-López M. Barrajón-Catalán E. Micol V. (2023). Hallmarks and biomarkers of skin senescence: an updated review of skin senotherapeutics. Antioxidants 12, 444. 10.3390/antiox12020444 36830002 PMC9952625

[B7] Cai Y. S. Zhou H. H. Zhu Y. H. Sun Q. Ji Y. Xue A. (2020). Elimination of senescent cells by β-galactosidase-targeted prodrug attenuates inflammation and restores physical function in aged mice. Cell Res. 30, 574–589. 10.1038/s41422-020-0314-9 32341413 PMC7184167

[B8] Cappuzzello C. Doni A. Dander E. Pasqualini F. Nebuloni M. Bottazzi B. (2016). Mesenchymal stromal cell-derived PTX3 promotes wound healing via fibrin remodeling. J. Invest. Dermatol. 136, 293–300. 10.1038/JID.2015.346 26763449

[B9] Choi K. Y. Sultan M. T. Ajiteru O. Hong H. Lee Y. J. Lee J. S. (2021). Treatment of fungal-infected diabetic wounds with low temperature plasma. Biomedicines 10, 27. 10.3390/biomedicines10010027 35052706 PMC8773309

[B10] Conway K. Ruge F. Price P. Harding K. G. Jiang W. G. (2007). Hepatocyte growth factor regulation: an integral part of why wounds become chronic. Wound Repair Regen. 15, 683–692. 10.1111/j.1524-475X.2007.00296.x 17971014

[B11] Ding X. L. Kakanj P. Leptin M. Eming S. A. (2021). Regulation of the wound healing response during aging. J. Invest. Dermatol. 141, 1063–1070. 10.1016/j.jid.2020.11.014 33558058

[B12] Duarte S. Panariello B. H. D. (2020). Comprehensive biomedical applications of low temperature plasmas. Arch. Biochem. Biophys. 693, 108560. 10.1016/j.abb.2020.108560 32857998 PMC7448743

[B13] Dubey S. K. Parab S. Alexander A. Agrawal M. Achalla V. P. K. Pal U. N. (2022). Cold atmospheric plasma therapy in wound healing. Process Biochem. 112, 112–123. 10.1016/j.procbio.2021.11.017

[B14] Dubuc A. Monsarrat P. Virard F. Merbahi N. Sarrette J.-P. Laurencin-Dalicieux S. (2018). Use of cold-atmospheric plasma in oncology: a concise systematic review. Ther. Adv. Med. Oncol. 10, 1758835918786475. 10.1177/1758835918786475 30046358 PMC6055243

[B15] Dungan C. M. Peck B. D. Walton R. G. Huang Z. Y. Bamman M. M. Kern P. A. (2020). *In vivo* analysis of γH2AX+ cells in skeletal muscle from aged and obese humans. FASEB J. 34, 7018–7035. 10.1096/fj.202000111RR 32246795 PMC7243467

[B16] Eming S. A. Martin P. Tomic-Canic M. (2014). Wound repair and regeneration: mechanisms, signaling, and translation. Sci. Transl. Med. 6, 265sr6. 10.1126/scitranslmed.3009337 25473038 PMC4973620

[B17] Ersanli C. Tzora A. Voidarou C. Skoufos S. Zeugolis D. I. Skoufos I. (2023). Biodiversity of skin microbiota as an important biomarker for wound healing. Biology 12, 1187. 10.3390/biology12091187 37759587 PMC10525143

[B18] Fujiwara T. Duscher D. Rustad K. C. Kosaraju R. Rodrigues M. Whittam A. J. (2016). Extracellular superoxide dismutase deficiency impairs wound healing in advanced age by reducing neovascularization and fibroblast function. Exp. Dermatol. 25, 206–211. 10.1111/exd.12909 26663425 PMC4998179

[B19] Fyhrquist N. Ruokolainen L. Suomalainen A. Lehtimäki S. Veckman V. Vendelin J. (2014). Acinetobacter species in the skin microbiota protect against allergic sensitization and inflammation. J. Allergy Clin. Immunol. 134, 1301–1309.e11. 10.1016/j.jaci.2014.07.059 25262465

[B20] Gan L. Duan J. Zhang S. Liu X. Poorun D. Liu X. (2019). Cold atmospheric plasma ameliorates imiquimod-induced psoriasiform dermatitis in mice by mediating antiproliferative effects. Free Radic. Res. 53, 269–280. 10.1080/10715762.2018.1564920 30663913

[B21] Gao J. Wang L. Y. Xia C. K. Yang X. Y. Cao Z. C. Zheng L. (2019). Cold atmospheric plasma promotes different types of superficial skin erosion wounds healing. Int. Wound J. 16, 1103–1111. 10.1111/iwj.13161 31207094 PMC7949367

[B22] Gharbia F. Z. Abouhashem A. S. Moqidem Y. A. Elbaz A. A. Abdellatif A. Singh K. (2023). Adult skin fibroblast state change in murine wound healing. Sci. Rep. 13, 886. 10.1038/s41598-022-27152-4 36650180 PMC9845335

[B23] Gonzalez A. C. D. O. Costa T. F. Andrade Z. D. A. Medrado A. R. A. P. (2016). Wound healing - a literature review. An. Bras. Dermatol. 91, 614–620. 10.1590/abd1806-4841.20164741 27828635 PMC5087220

[B24] Hasse S. Duong Tran T. Hahn O. Kindler S. Metelmann H.-R. Von Woedtke T. (2016). Induction of proliferation of basal epidermal keratinocytes by cold atmospheric-pressure plasma. Clin. Exp. Dermatol. 41, 202–209. 10.1111/ced.12735 26175125

[B25] Hoffmann C. Berganza C. Zhang J. (2013). Cold Atmospheric Plasma: methods of production and application in dentistry and oncology. Med. Gas. Res. 3, 21. 10.1186/2045-9912-3-21 24083477 PMC4016545

[B26] Hosseini M. Koehler K. R. Shafiee A. (2022). Biofabrication of human skin with its appendages. Adv. Healthc. Mat. 11, 2201626. 10.1002/adhm.202201626 PMC11469047 36063498

[B27] Isbary G. Shimizu T. Li Y.-F. Stolz W. Thomas H. M. Morfill G. E. (2013). Cold atmospheric plasma devices for medical issues. Expert Rev. Med. Devices 10, 367–377. 10.1586/erd.13.4 23668708

[B28] Ishaq M. Bazaka K. Ostrikov K. (2015). Intracellular effects of atmospheric-pressure plasmas on melanoma cancer cells. Phys. Plasmas 22, 122003. 10.1063/1.4933366

[B29] Ji M. C. Li J. Y. Wang Y. Li F. Y. Man J. Li J. (2022). Advances in chitosan-based wound dressings: modifications, fabrications, applications and prospects. Carbohydr. Polym. 297, 120058. 10.1016/j.carbpol.2022.120058 36184154

[B30] Karthik C. Sarngadharan S. C. Thomas V. (2023). Low-temperature plasma techniques in biomedical applications and therapeutics: an overview. Int. J. Mol. Sci. 25, 524. 10.3390/ijms25010524 38203693 PMC10779006

[B31] Kataoka H. Kawaguchi M. Fukushima T. Shimomura T. (2018). Hepatocyte growth factor activator inhibitors (HAI‐1 and HAI‐2): emerging key players in epithelial integrity and cancer. Pathol. Int. 68, 145–158. 10.1111/pin.12647 29431273

[B32] Kim D. E. Dollé M. E. T. Vermeij W. P. Gyenis A. Vogel K. Hoeijmakers J. H. J. (2020a). Deficiency in the DNA repair protein ERCC1 triggers a link between senescence and apoptosis in human fibroblasts and mouse skin. Aging Cell 19, e13072. 10.1111/acel.13072 31737985 PMC7059167

[B33] Kim J. H. Ruegger P. R. Lebig E. G. VanSchalkwyk S. Jeske D. R. Hsiao A. (2020b). High levels of oxidative stress create a microenvironment that significantly decreases the diversity of the microbiota in diabetic chronic wounds and promotes biofilm formation. Front. Cell. Infect. Microbiol. 10, 259. 10.3389/fcimb.2020.00259 32582564 PMC7283391

[B34] Knoedler S. Knoedler L. Kauke-Navarro M. Rinkevich Y. Hundeshagen G. Harhaus L. (2023). Regulatory T cells in skin regeneration and wound healing. Mil. Med. Res. 10, 49. 10.1186/s40779-023-00484-6 37867188 PMC10591349

[B35] Kogelschatz U. (2003). Dielectric-barrier discharges: their history, discharge physics, and industrial applications. Plasma Chem. Plasma Process. 23, 1–46. 10.1023/A:1022470901385

[B36] Kou Z. Li B. Aierken A. Tan N. Li C. Han M. (2023). Mesenchymal stem cells pretreated with collagen promote skin wound-healing. Int. J. Mol. Sci. 24, 8688. 10.3390/ijms24108688 37240027 PMC10218687

[B37] Las Heras K. Igartua M. Santos-Vizcaino E. Hernandez R. M. (2020). Chronic wounds: current status, available strategies and emerging therapeutic solutions. J. Control. Release 328, 532–550. 10.1016/j.jconrel.2020.09.039 32971198

[B38] Li S. Y. Sun J. C. Yang J. X. Yang Y. Ding H. F. Yu B. (2023). Gelatin methacryloyl (GelMA) loaded with concentrated hypoxic pretreated adipose-derived mesenchymal stem cells(ADSCs) conditioned medium promotes wound healing and vascular regeneration in aged skin. Biomater. Res. 27, 11. 10.1186/s40824-023-00352-3 36782342 PMC9926638

[B39] Liang Y. Q. Liang Y. P. Zhang H. L. Guo B. L. (2022). Antibacterial biomaterials for skin wound dressing. Asian J. Pharm. Sci. 17, 353–384. 10.1016/j.ajps.2022.01.001 35782328 PMC9237601

[B40] Liao F. Y. Chen L. Luo P. Jiang Z. Y. Chen Z. L. Wang Z. (2020). PC4 serves as a negative regulator of skin wound healing in mice. Burns Trauma 8, tkaa010. 10.1093/burnst/tkaa010 32373645 PMC7198317

[B41] Liu W. J. Yan F. Xu Z. Y. Chen Q. Y. Ren J. Wang Q. (2022). Urolithin A protects human dermal fibroblasts from UVA-induced photoaging through NRF2 activation and mitophagy. J. Photochem. Photobiol. B 232, 112462. 10.1016/j.jphotobiol.2022.112462 35567884

[B42] Luo R. Z. Dai J. Y. Zhang J. P. Li Z. (2021). Accelerated skin wound healing by electrical stimulation. Adv. Healthc. Mat. 10, 2100557. 10.1002/adhm.202100557 33945225

[B43] Ma L. Chen Y. Gong Q. Cheng Z. Ran C. F. Liu K. (2023). Cold atmospheric plasma alleviates radiation-induced skin injury by suppressing inflammation and promoting repair. Free Radic. Biol. Med. 204, 184–194. 10.1016/j.freeradbiomed.2023.05.002 37172912

[B44] Manchanda M. Torres M. Inuossa F. Bansal R. Kumar R. Hunt M. (2023). Metabolic reprogramming and reliance in human skin wound healing. J. Invest. Dermatol. 143, 2039–2051.e10. 10.1016/j.jid.2023.02.039 37061123

[B45] Martusevich A. K. Surovegina A. V. Bocharin I. V. Nazarov V. V. Minenko I. A. Artamonov M.Yu (2022). Cold argon athmospheric plasma for biomedicine: biological effects, applications and possibilities. Antioxidants 11, 1262. 10.3390/antiox11071262 35883753 PMC9311881

[B46] Mei F. F. Liu J. J. Wu J. T. Duan Z. W. Chen M. X. Meng K. (2020). Collagen peptides isolated from *Salmo salar* and *Tilapia nilotica* skin accelerate wound healing by altering cutaneous microbiome colonization via upregulated NOD2 and BD14. J. Agric. Food Chem. 68, 1621–1633. 10.1021/acs.jafc.9b08002 31967468

[B47] Mi J. Xie C. Zeng L. Zhu Z. Chen N. He Q. (2022). Bacillus subtilis WB800N alleviates diabetic wounds in mice by regulating gut microbiota homeostasis and TLR2. J. Appl. Microbiol. 133, 436–447. 10.1111/jam.15547 35332963

[B48] Moeini A. Pedram P. Makvandi P. Malinconico M. Gomez d’Ayala G. (2020). Wound healing and antimicrobial effect of active secondary metabolites in chitosan-based wound dressings: a review. Carbohydr. Polym. 233, 115839. 10.1016/j.carbpol.2020.115839 32059889

[B49] Najafzadehvarzi H. Ghasemi M. Sohbatzadeh F. Aminjarrahi M. Darzi R. E. (2022). Risk assessment of a cold atmospheric physical argon plasma jet on the skin, liver, and biochemical factors in an animal model. Med. Eng. Phys. 106, 103826. 10.1016/j.medengphy.2022.103826 35926949

[B50] Nguyen D. B. Mok Y. S. Huynh D. L. Jeong D. K. Lee W. G. (2019). Application of plasma jet to the inhibition of the proliferation of hepatic malignant cells via reactive oxygen species generation. Plasma Process. Polym. 16, 1800173. 10.1002/ppap.201800173

[B51] Nguyen T. T. Mobashery S. Chang M. (2016). “Roles of matrix metalloproteinases in cutaneous wound healing,” in Wound healing - new insights into ancient challenges. Editor Alexandrescu V. A. (IntechOpen J.). 10.5772/64611

[B52] Niedźwiedź I. Waśko A. Pawłat J. Polak-Berecka M. (2019). The state of research on antimicrobial activity of cold plasma. Pol. J. Microbiol. 68, 153–164. 10.33073/pjm-2019-028 31250588 PMC7256829

[B53] Nourian Dehkordi A. Mirahmadi Babaheydari F. Chehelgerdi M. Raeisi Dehkordi S. (2019). Skin tissue engineering: wound healing based on stem-cell-based therapeutic strategies. Stem Cell Res. Ther. 10, 111. 10.1186/s13287-019-1212-2 30922387 PMC6440165

[B54] Presta M. Foglio E. Churruca Schuind A. Ronca R. (2018). Long pentraxin-3 modulates the angiogenic activity of fibroblast growth factor-2. Front. Immunol. 9, 2327. 10.3389/fimmu.2018.02327 30349543 PMC6187966

[B55] Quan T. H. (2023). Human skin aging and the anti-aging properties of retinol. Biomolecules 13, 1614. 10.3390/biom13111614 38002296 PMC10669284

[B56] Ramos T. N. Bullard D. C. Barnum S. R. (2014). ICAM-1: isoforms and phenotypes. J. Immunol. 192, 4469–4474. 10.4049/jimmunol.1400135 24795464 PMC4015451

[B57] Ranc A. Dubourg G. Fournier P. E. Raoult D. Fenollar F. (2018). Delftia tsuruhatensis, an emergent opportunistic healthcare-associated pathogen. Emerg. Infect. Dis. 24, 594–596. 10.3201/eid2403.160939 29460754 PMC5823324

[B58] Reuter S. Von Woedtke T. Weltmann K.-D. (2018). The kINPen—a review on physics and chemistry of the atmospheric pressure plasma jet and its applications. J. Phys. Appl. Phys. 51, 233001. 10.1088/1361-6463/aab3ad

[B59] Rezaie F. Momeni-Moghaddam M. Naderi-Meshkin H. (2019). Regeneration and repair of skin wounds: various strategies for treatment. Int. J. Low. Extrem. Wounds 18, 247–261. 10.1177/1534734619859214 31257948

[B60] Roy S. Santra S. Das A. Dixith S. Sinha M. Ghatak S. (2020). *Staphylococcus aureus* biofilm infection compromises wound healing by causing deficiencies in granulation tissue collagen. Ann. Surg. 271, 1174–1185. 10.1097/SLA.0000000000003053 30614873 PMC7065840

[B61] Ryan M. P. Sevjahova L. Gorman R. White S. (2022). The emergence of the genus comamonas as important opportunistic pathogens. Pathogens 11, 1032. 10.3390/pathogens11091032 36145464 PMC9504711

[B62] Sedik A. A. Salama M. Fathy K. Salama A. (2023). Cold plasma approach fortifies the topical application of thymoquinone intended for wound healing via up-regulating the levels of TGF-ß, VEGF, and α-SMA in rats. Int. Immunopharmacol. 122, 110634. 10.1016/j.intimp.2023.110634 37451012

[B63] Sibai M. Altuntaş E. Yıldırım B. Öztürk G. Yıldırım S. Demircan T. (2020). Microbiome and longevity: high abundance of longevity-linked Muribaculaceae in the gut of the long-living rodent spalax leucodon. OMICS J. Integr. Biol. 24, 592–601. 10.1089/omi.2020.0116 32907488

[B64] Sohn K. M. Baek J.-Y. (2015). Delftia lacustris septicemia in a pheochromocytoma patient: case report and literature review. Infect. Dis. 47, 349–353. 10.3109/00365548.2014.993422 25712727

[B65] Subramaniam T. Fauzi M. B. Lokanathan Y. Law J. X. (2021). The role of calcium in wound healing. Int. J. Mol. Sci. 22, 6486. 10.3390/ijms22126486 34204292 PMC8235376

[B66] Sun J. C. Liu X. Z. Shen C. Zhang W. Niu Y. Z. (2021). Adiponectin receptor agonist AdipoRon blocks skin inflamm‐ageing by regulating mitochondrial dynamics. Cell Prolif. 54, e13155. 10.1111/cpr.13155 34725875 PMC8666283

[B67] Tomic-Canic M. Burgess J. L. O’Neill K. E. Strbo N. Pastar I. (2020). Skin microbiota and its interplay with wound healing. Am. J. Clin. Dermatol. 21, 36–43. 10.1007/s40257-020-00536-w 32914215 PMC7584558

[B68] Wang C.-H. Cherng J.-H. Liu C.-C. Fang T.-J. Hong Z.-J. Chang S.-J. (2021a). Procoagulant and antimicrobial effects of chitosan in wound healing. Int. J. Mol. Sci. 22, 7067. 10.3390/ijms22137067 34209202 PMC8269297

[B69] Wang Y. Wu Y. Long L. Y. Yang L. Fu D. H. Hu C. (2021b). Inflammation-responsive drug-loaded hydrogels with sequential hemostasis, antibacterial, and anti-inflammatory behavior for chronically infected diabetic wound treatment. ACS Appl. Mat. Interfaces 13, 33584–33599. 10.1021/acsami.1c09889 34240605

[B70] Weinheimer-Haus E. M. Mirza R. E. Koh T. J. (2015). Nod-like receptor protein-3 inflammasome plays an important role during early stages of wound healing. PLoS ONE 10, e0119106. 10.1371/journal.pone.0119106 25793779 PMC4368510

[B71] Wu Y. J. Yu S. Y. Zhang X. Y. Wang X. Z. Zhang J. J. (2023). The regulatory mechanism of cold plasma in relation to cell activity and its application in biomedical and animal husbandry practices. Int. J. Mol. Sci. 24, 7160. 10.3390/ijms24087160 37108320 PMC10138629

[B72] Yu R. Zhang H. L. Guo B. L. (2022). Conductive biomaterials as bioactive wound dressing for wound healing and skin tissue engineering. Nano-Micro Lett. 14, 1. 10.1007/s40820-021-00751-y PMC8639891 34859323

[B73] Zhang J. H. Yan Y. Li Y. J. Shen C. C. Zhang Y. M. (2021). Topical effect of benzalkonium bromide on wound healing and potential cellular and molecular mechanisms. Int. Wound J. 18, 566–576. 10.1111/iwj.13555 33512783 PMC8450784

[B74] Zhang Y. Wang S. Q. Yang Y. X. Zhao S. You J. H. Wang J. (2023). Scarless wound healing programmed by core-shell microneedles. Nat. Commun. 14, 3431. 10.1038/s41467-023-39129-6 37301874 PMC10257705

[B75] Zhao R. L. Liang H. Clarke E. Jackson C. Xue M. L. (2016). Inflammation in chronic wounds. Int. J. Mol. Sci. 17, 2085. 10.3390/ijms17122085 27973441 PMC5187885

[B76] Zhou C. C. Zhang B. Y. Yang Y. Q. Jiang Q. Li T. Y. Gong J. (2023). Stem cell-derived exosomes: emerging therapeutic opportunities for wound healing. Stem Cell Res. Ther. 14, 107. 10.1186/s13287-023-03345-0 37101197 PMC10134577

[B77] Zhou J. Dong Y. B. Liu J. L. Ren J. Wu J. Y. Zhu N. W. (2020). AQP5 regulates the proliferation and differentiation of epidermal stem cells in skin aging. Braz. J. Med. Biol. Res. 53, e10009. 10.1590/1414-431x202010009 32965322 PMC7510230

[B78] Zulkefli N. Che Zahari C. N. M. Sayuti N. H. Kamarudin A. A. Saad N. Hamezah H. S. (2023). Flavonoids as potential wound-healing molecules: emphasis on pathways perspective. Int. J. Mol. Sci. 24, 4607. 10.3390/ijms24054607 36902038 PMC10003005

